# Modeling buprenorphine reduction of fentanyl-induced respiratory depression

**DOI:** 10.1172/jci.insight.156973

**Published:** 2022-05-09

**Authors:** Erik Olofsen, Marijke Hyke Algera, Laurence Moss, Robert L. Dobbins, Geert J. Groeneveld, Monique van Velzen, Marieke Niesters, Albert Dahan, Celine M. Laffont

**Affiliations:** 1 Department of Anesthesiology, Leiden University Medical Center, Leiden, Netherlands.; 2 Centre for Human Drug Research, Leiden, Netherlands.; 3 Global Medicines Development, Indivior Inc., North Chesterfield, Virginia, USA.

**Keywords:** Clinical Trials, Neuroscience, Addiction, Anesthesiology, Pharmacology

## Abstract

**BACKGROUND:**

Potent synthetic opioids, such as fentanyl, are increasingly abused, resulting in unprecedented numbers of fatalities from respiratory depression. Treatment with the high-affinity mu-opioid receptor partial agonist buprenorphine may prevent fatalities by reducing binding of potent opioids to the opioid receptor, limiting respiratory depression.

**METHODS:**

To characterize buprenorphine-fentanyl interaction at the level of the mu-opioid receptor in 2 populations (opioid-naive individuals and individuals who chronically use high-dose opioids), the effects of escalating i.v. fentanyl doses with range 0.075–0.35 mg/70 kg (opioid naive) and 0.25–0.70 mg/70 kg (chronic opioid use) on iso-hypercapnic ventilation at 2–3 background doses of buprenorphine (target plasma concentrations range: 0.2–5 ng/mL) were quantified using receptor association/dissociation models combined with biophase distribution models.

**RESULTS:**

Buprenorphine produced mild respiratory depression, while high doses of fentanyl caused pronounced respiratory depression and apnea in both populations. When combined with fentanyl, buprenorphine produced a receptor binding–dependent reduction of fentanyl-induced respiratory depression in both populations. In individuals with chronic opioid use, at buprenorphine plasma concentrations of 2 ng/mL or higher, a protective effect against high-dose fentanyl was observed.

**CONCLUSION:**

Overall, the results indicate that when buprenorphine mu-opioid receptor occupancy is sufficiently high, fentanyl is unable to activate the mu-opioid receptor and consequently will not cause further respiratory depression in addition to the mild respiratory effects of buprenorphine.

**TRIAL REGISTRATION:**

Trialregister.nl, no. NL7028 (https://www.trialregister.nl/trial/7028)

**FUNDING:**

Indivior Inc., North Chesterfield, Virginia, USA.

## Introduction

Over the past 2 decades, abuse of illicit or prescribed opioids has caused a soaring number of fatalities from opioid-induced respiratory depression (1, 2). According to the CDC, around 76,000 people in the United States died from an opioid overdose in the 12 months ending in June 2021 (3). The majority of these deaths are the result of synthetic opioids such as fentanyl being ingested as a substitute for heroin or with psychostimulants (such as cocaine and methamphetamine) that had been adulterated with the opioid (2). This major health and socioeconomic burden to society requires immediate and ongoing attention. One option to reduce the risk of a fatal respiratory depression from an opioid overdose in patients with an opioid use disorder is treatment with buprenorphine (4, 5). Buprenorphine is a semisynthetic opioid derived from the morphine precursor thebaine and is a mu-opioid receptor (MOR) partial agonist (6–8). We previously showed that the high affinity of buprenorphine at the MOR, which is directly related to its slow receptor kinetics, makes reversal of buprenorphine effects difficult with the opioid antagonist naloxone, even at high naloxone doses (7). Subsequent pharmacokinetic/pharmacodynamic modeling studies suggest that buprenorphine at sufficiently high plasma concentrations prevents binding of potent opioids to the MOR, causing less respiratory depression and other opioid-related unwanted effects, including opioid craving (8, 9). However, this requires experimental proof. In the current pharmacokinetic/pharmacodynamic modeling study, we examined the interaction of buprenorphine and the potent opioid fentanyl on ventilation in opioid-naive volunteers and individuals with chronic opioid use. The main goal of this study was to characterize buprenorphine-fentanyl interactions at the MOR and to determine buprenorphine plasma levels needed to protect against the respiratory depression induced by fentanyl in individuals with chronic opioid use.

## Results

**Figure 1:**
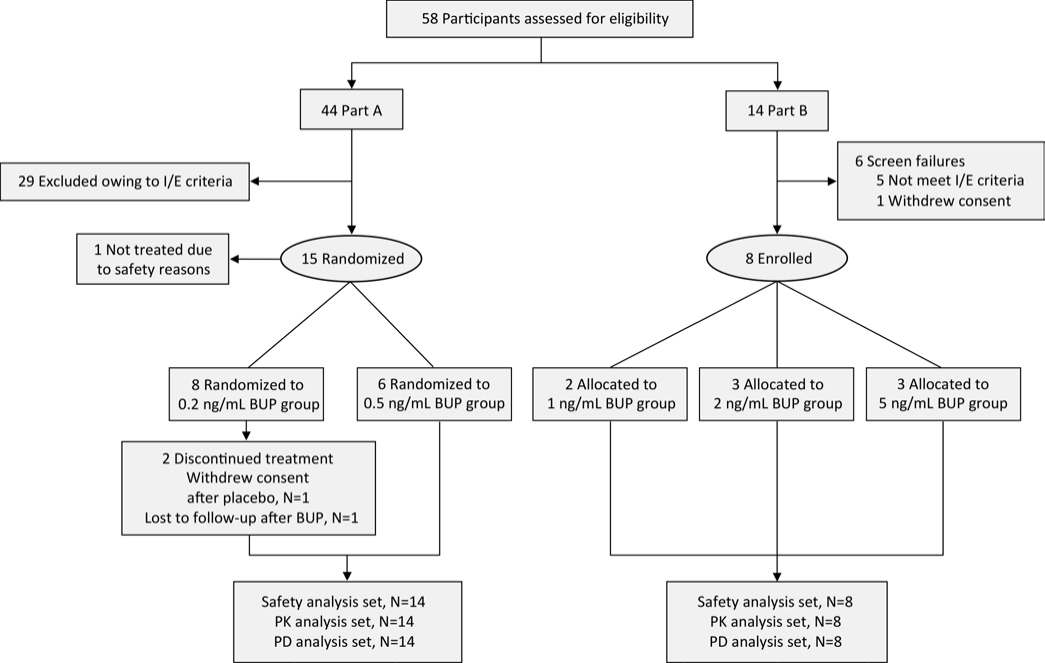
CONSORT flow diagram. BUP, buprenorphine; I/E, inclusion/exclusion; PD, pharmacodynamic; PK, pharmacokinetic.

The study was performed in 2 parts: part A was conducted in 14 opioid-naive volunteers (7 men/7 women, on average 24 years old) and part B was conducted in 8 individuals with chronic opioid use (3 men/5 women, on average 42 years old) (Figure 1). Both parts included 2 study periods (period 1 and period 2), during which participants received continuous i.v. infusion of buprenorphine or a placebo coadministered with up to 4 escalating fentanyl i.v. doses (Table 1). Opioid-naive volunteers had the option to participate in a third study period (period 3) where they received an i.v. infusion of buprenorphine alone. Two of the opioid-naive volunteers only participated to period 1 (1 volunteer withdrew consent after completion of the first experimental session; the other did not return for unknown reasons); available data of both individuals were included in the analyses (i.e., 1 placebo/fentanyl data set and 1 buprenorphine/fentanyl data set). Five volunteers participated in period 3. All individuals with chronic opioid use completed periods 1 and 2. They had used on average 220 ± 134 mg morphine equivalents per day for an average of 7 years (range: 3 months to 29 years) and were transitioned to 114 mg oxycodone per day (range: 50–315 mg) for the study, with the last dose of oxycodone given at least 15 hours prior to each period. Their opioid use was related to chronic pain (*n* = 6) or opioid use disorder (*n* = 2).

**Figure 2:**
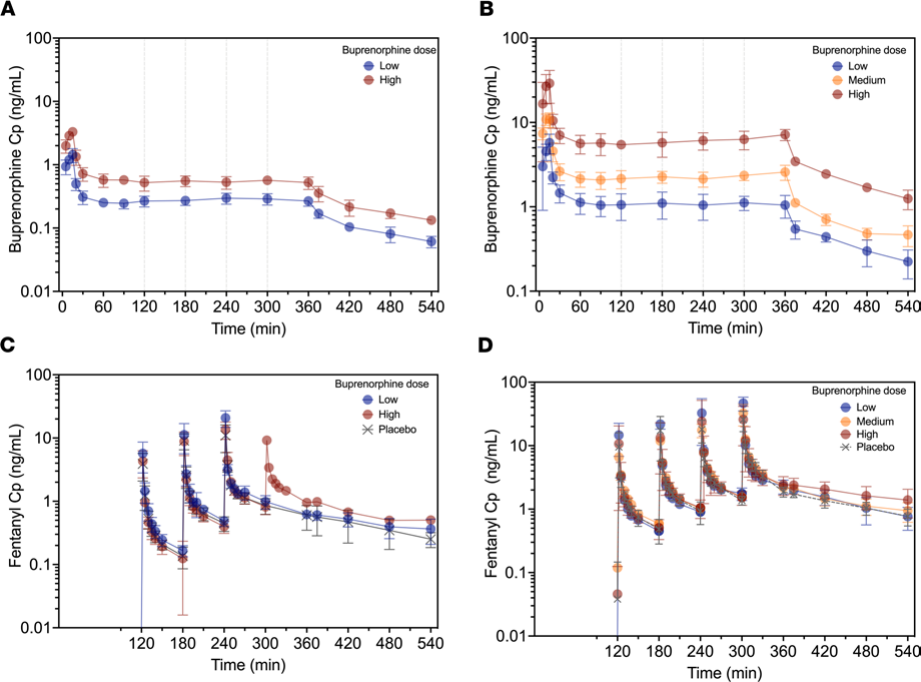
Mean ± SD pharmacokinetic profiles. Buprenorphine plasma concentrations (Cp) in opioid-naive volunteers receiving low- or high-dose buprenorphine targeting a plasma concentration of 0.2 or 0.5 ng/mL, respectively (**A**) and individuals with chronic opioid use receiving low-, medium-, or high-dose buprenorphine targeting a plasma concentration of 1, 2, or 5 ng/mL, respectively (**B**). Fentanyl plasma concentrations (Cp) in opioid-naive volunteers (**C**) and individuals with chronic opioid use (**D**) receiving multiple doses of fentanyl across treatment groups (opioid-naive: 0.075, 0.15, 0.25, and 0.35 mg/70 kg; chronic opioid use: 0.25, 0.35, 0.50, and 0.70 mg/70 kg).

All study participants (opioid-naive volunteers and those with chronic opioid use) completed their experimental sessions without any unexpected adverse events (see ref. 10 for details on safety findings). Eight opioid-naive volunteers received low-dose buprenorphine (target plasma concentration: 0.2 ng/mL), 6 others high-dose buprenorphine (target concentration: 0.5 ng/mL), with matching measured plasma concentrations (mean ± SD) of 0.28 ± 0.05 and 0.54 ± 0.08 ng/mL, respectively (Figure 2). Individuals with chronic opioid use received low-dose (*n* = 2, target concentration: 1 ng/mL), medium-dose (*n* = 3, target concentration: 2 ng/mL), or high-dose (*n* = 3, target concentration: 5 ng/mL) buprenorphine, with matching measured plasma concentrations of 1.08 ± 0.33, 2.28 ± 0.40, and 6.12 ± 1.26 ng/mL, respectively (Figure 2). Because of the occurrences of apneic events, the number of fentanyl doses was restricted to either 2 or 3 (irrespective of treatment) in 13/14 opioid-naive volunteers with just 1 individual receiving all 4 fentanyl doses at high-dose buprenorphine (Figure 2). In individuals with chronic opioid use, all 4 doses were given during the buprenorphine session but 2–4 doses during the placebo session.

### Population pharmacokinetic analyses.

**Figure 3:**
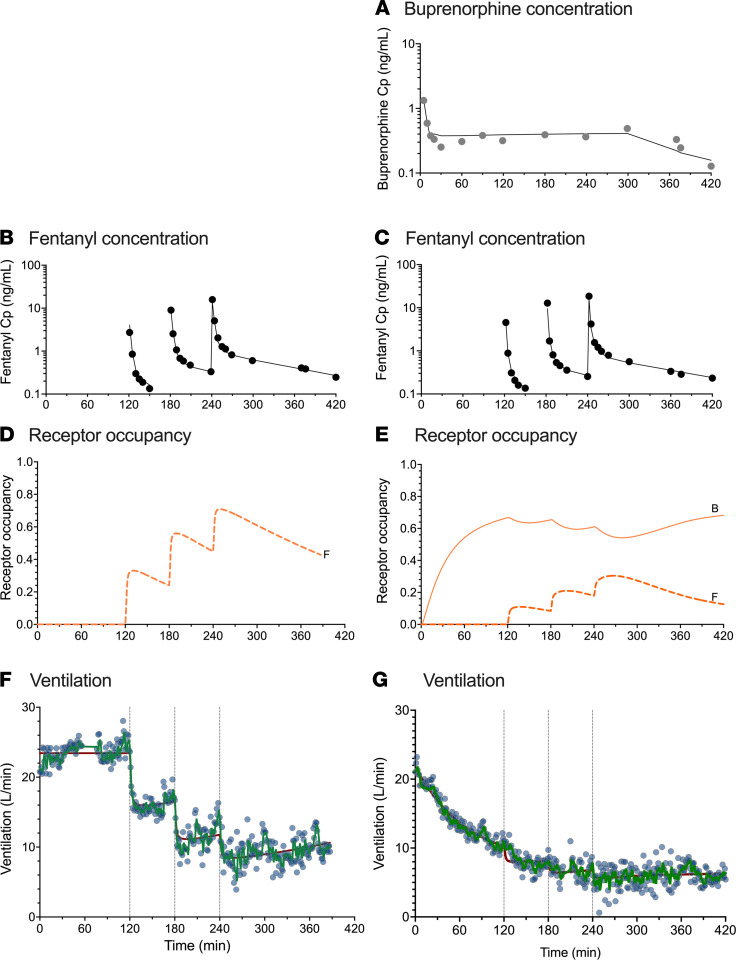
Example of data results and analyses of an opioid-naive individual. Data fits and predicted receptor occupancy driving the effect of fentanyl (ascending doses of 0.075, 0.15, and 0.25 mg/70 kg) on minute ventilation at the background of placebo infusion (**B**, **D**, and **F**) and high-dose buprenorphine infusion targeting a plasma concentration of 0.5 ng/mL (**A**, **C**, **E**, and **G**) in an opioid-naive volunteer. (**A**) Measured buprenorphine plasma concentration (Cp) (gray circles) and data fits (continuous line). (**B** and **C**) Measured fentanyl plasma concentration (Cp) (black circles) and data fits (continuous lines). (**D** and **E**) Predicted receptor occupancy for fentanyl (broken line, **F**) and buprenorphine (continuous line, **B**). (**F** and **G**) Measured ventilation (blue circles) and data fit of the model with a Kalman filter (green line) and data fit of the model without a Kalman filter (red line). Acquisition of ventilation data was sometimes interrupted for various reasons (see text); in this case because the individual had to urinate (**F** and **G**).

**Figure 4:**
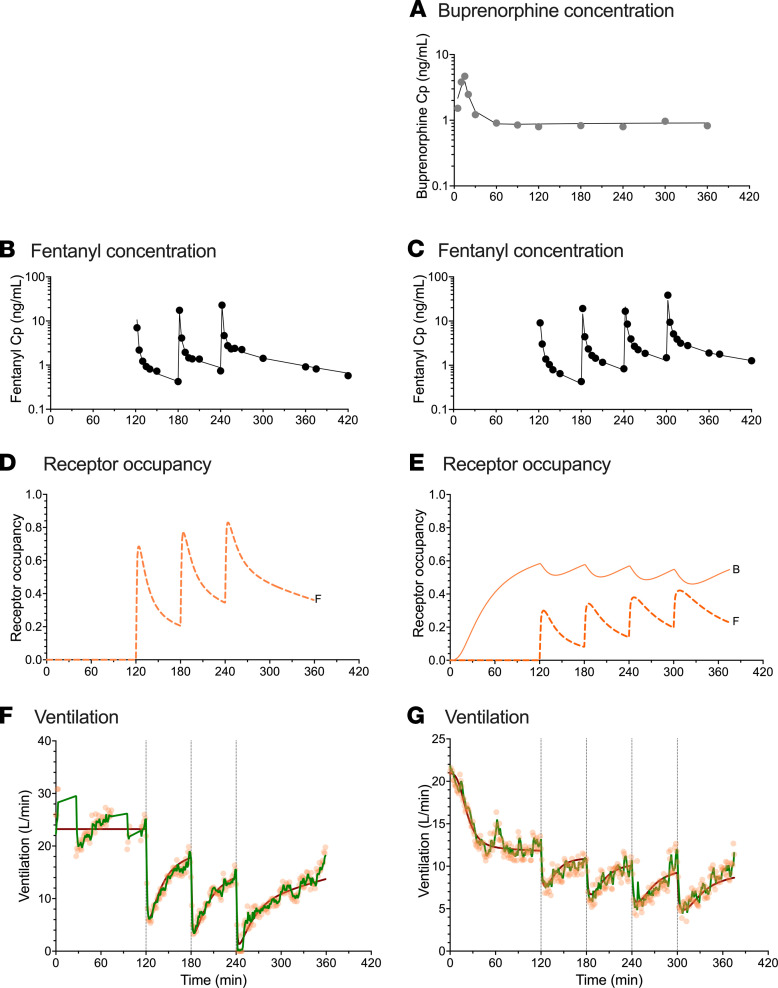
Example of data results and analyses of an individual with chronic opioid use. Data fits and predicted receptor occupancy driving the effect of fentanyl (ascending doses of 0.25, 0.35, 0.50, and 0.70 mg/70 kg) on minute ventilation at the background of placebo infusion (**B**, **D**, and **F**) and low-dose buprenorphine infusion targeting a plasma concentration of 1 ng/mL (**A**, **C**, **E**, and **G**) in an individual with chronic opioid use. (**A**) Measured buprenorphine plasma concentration (Cp) (gray circles) and data fits (continuous line). (**B** and **C**) Measured fentanyl plasma concentration (Cp) (black circles) and data fits (continuous lines). (**D** and **E**) Predicted receptor occupancy for fentanyl (broken line, **F**) and buprenorphine (continuous line, **B**). (**F** and **G**) Measured ventilation (orange circles) and data fit of the model with a Kalman filter (green line) and data fit of the model without a Kalman filter (red line). Acquisition of ventilation data was sometimes interrupted for various reasons; in this case because the individual had to urinate (**F**).

Three-compartment models best described buprenorphine and fentanyl plasma concentration data. Concentrations were log-transformed for the analysis and an additive error model was retained in each case (which can be interpreted as a proportional error model on untransformed data). The parameter estimates of the final pharmacokinetic models are given in Table 2. No significant differences between the 2 populations were identified during the covariate analysis. Goodness-of-fit plots are given in Supplemental Figure 1; supplemental material available online with this article; https://doi.org/10.1172/jci.insight.156973DS1 and prediction- and variability-corrected visual predictive checks (pvcVPCs), comparing observations with model predictions, are given in Supplemental Figures 2 and 3. Examples of data fits are given in Figure 3 and Figure 4. Overall, the inspection of goodness-of-fit plots and pvcVPCs indicated that the models well described the concentration data for both buprenorphine and fentanyl.

### Population pharmacokinetic/pharmacodynamic analyses.

**Figure 5:**
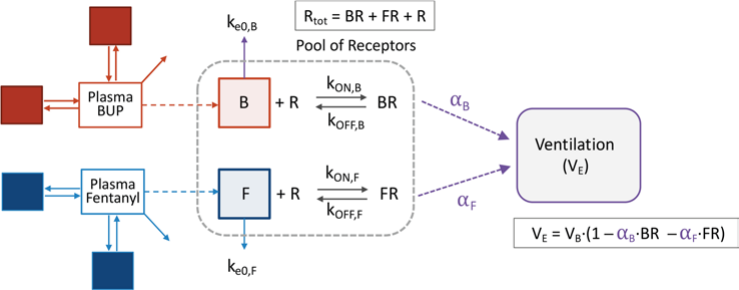
Integrated pharmacokinetic/pharmacodynamic model. α_B_, α_F_: intrinsic activity of buprenorphine and fentanyl, respectively (which also accounts for receptor reserve); B, F: effect site concentrations for buprenorphine and fentanyl, respectively; BR, FR: concentrations of receptors bound to buprenorphine and fentanyl, respectively; BUP: buprenorphine; k_e0,B_, k_e0,F_: equilibration rate constants for buprenorphine and fentanyl, respectively; k_ON,B_, k_OFF,B_: buprenorphine association and disassociation rate constants; k_ON,F_, k_OFF,F_: fentanyl association and disassociation rate constants; R: concentration of unbound receptors; V_B_: baseline ventilation; V_E_: minute ventilation. Because fentanyl associates and dissociates rapidly from the receptors, C_50,F_ (=k_OFF,F_/k_ON,F_) was estimated in place of k_OFF,F_ and k_ON,F_.

Minute ventilation data measured in opioid-naive volunteers and those with chronic opioid use were modeled simultaneously using a population pharmacokinetic/pharmacodynamic modeling approach. The integrated pharmacokinetic/pharmacodynamic model, incorporating receptor association/dissociation models with biophase distribution models, is represented in Figure 5. Because fentanyl associates and dissociates rapidly from MORs, C_50F_ (= k_OFF,F_/k_ON,F_) was estimated in place of fentanyl association and dissociation rate constants k_ON,F_ and k_OFF,F_.

**Figure 6:**
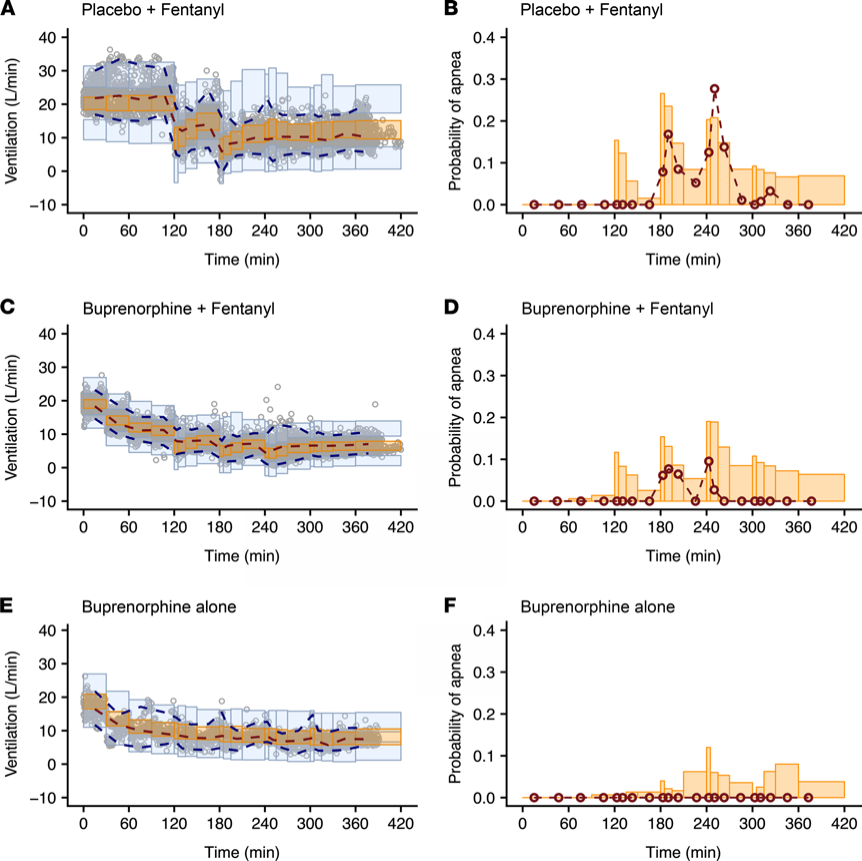
Prediction- and variability-corrected visual predictive checks of the pharmacodynamic model with a Kalman filter in opioid-naive individuals for the various drug administrations and probabilities of apnea for the same conditions. (**A** and **B**) Fentanyl given at the background of placebo infusion. (**C** and **D**) Fentanyl given at the background of buprenorphine infusion. (**E** and **F**) Just buprenorphine. The dots in **A**, **C**, and **E** are the 1-minute ventilation averages; the broken lines are the observed percentiles (dark orange: median, dark blue: 2.5th and 97.5th percentiles); the bins are the 95% confidence intervals of simulated percentiles (orange bins: median, blue bins: 2.5th and 97.5th percentiles). The right panels (**B**, **D**, and **F**) give the probability of apnea. The red symbols are the probabilities of the observed apneic episodes; the orange bins are the simulated 95% confidence intervals of the probability of apnea.

**Figure 7:**
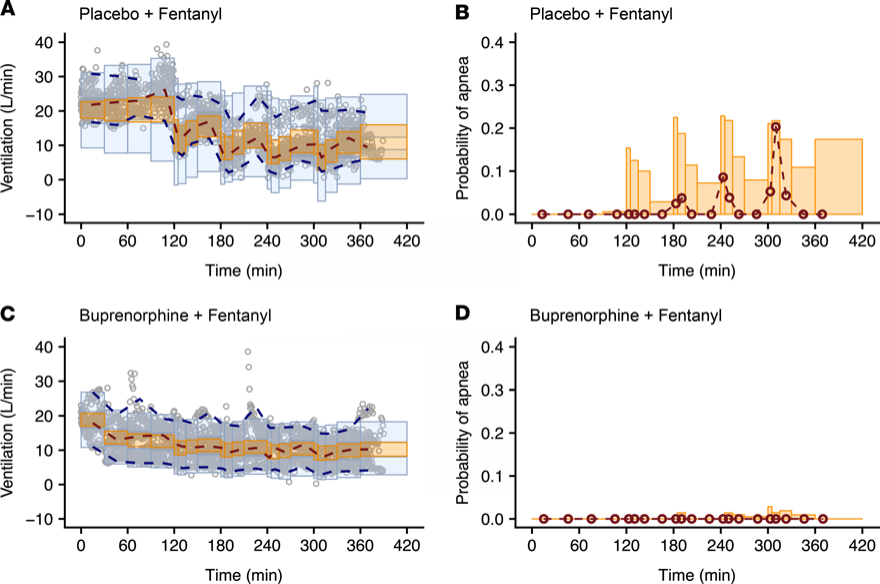
Prediction- and variability-corrected visual predictive checks of the pharmacodynamic model with a Kalman filter in individuals with chronic opioid use for the various drug administrations and probabilities of apnea for the same conditions. (**A** and **B**) Fentanyl given at the background of placebo infusion. (**C** and **D**) Fentanyl given at the background of buprenorphine infusion. The dots in **A** and **C** are the 1-minute ventilation averages; the broken lines are the observed percentiles (dark orange: median, dark blue: 2.5th and 97.5th percentiles); the bins are the 95% confidence intervals of simulated percentiles (orange bins: median, blue bins: 2.5th and 97.5th percentiles). The right panels (**B** and **D**) give the probability of apnea. The red symbols are the probabilities of the observed apneic episodes; the orange bins are the simulated 95% confidence intervals of the probability of apnea.

A Kalman filter was implemented to account for the correlations in residual noise (i.e., measurement noise). Typically, pharmacokinetic/pharmacodynamic models assume that the residual error is uncorrelated. However, with ventilation measurements every minute, correlations between residuals are likely to occur and can affect the estimation of model parameters if not appropriately accounted for. The parameter estimates of the final models with and without implementation of a Kalman filter are given in Table 3 and Supplemental Table 1, respectively. Upon model comparison, the model without the Kalman filter displayed an overprediction of the variability and a misprediction of the probability of apnea (see Figure 6 and Figure 7 compared with Supplemental Figures 4 and 5). Additionally, the model without the Kalman filter produced correlated noise when examining the autocorrelation and cross-correlation functions. This is exemplified in Supplemental Figure 6, which shows the autocorrelation function of the residuals for an analysis without (blue line) and with the Kalman filter (black line). The function shows “white” noise for residuals when a parallel noise component was added to the structural model, while they were correlated in the model without such a component. Since the model with the Kalman filter addressed these issues satisfactorily, it was considered the final model.

The analyses revealed several significant differences between opioid-naive volunteers and those with chronic opioid use in terms of buprenorphine association rate constant k_ON,B_ (opioid naive: 0.26 ± 0.02 mL.ng^–1^.min^–1^ versus chronic opioid use: 0.085 ± 0.009 mL.ng^–1^.min^–1^), buprenorphine-intrinsic activity α_B_ (opioid naive: 0.81 ± 0.03 versus chronic opioid use: 0.48 ± 0.03), and fentanyl potency C_50,F_ (opioid naive: 0.68 ± 0.13 ng/mL versus chronic opioid use: 2.23 ± 0.53 ng/mL) (see Table 3). These results suggest differences in receptor kinetics between the 2 populations, with a lower buprenorphine and fentanyl sensitivity in individuals with chronic opioid use compared with opioid-naive volunteers. In both populations, α_B_ was less than 1 while α_F_ was fixed to 1 with no associated variability, suggesting that apnea occurs at infinite fentanyl concentrations. However, the use of the Kalman filter allowed adequate prediction of apnea (Figures 6 and 7). In opioid-naive volunteers, differences in the magnitude of residual noise were observed between study occasions, with less residual noise when buprenorphine was given compared with a placebo (Table 3).

Examples of data fits are given in Figures 3 and 4 for 1 opioid-naive volunteer and 1 individual with chronic opioid use, respectively. They show that the model with the Kalman filter (green lines in Figure 3, F and G, and Figure 4, F and G) well described the minute ventilation data. Also, the output of the model without the Kalman filter is included (red lines in Figure 3, F and G, and Figure 4, F and G). Goodness-of-fit plots are given in Supplemental Figure 1, G–I. The pvcVPCs, comparing observations with model predictions, are given in Figure 6 (opioid naive) and Figure 7 (chronic opioid use) for the model with the Kalman filter and in Supplemental Figures 4 and 5 for the model without the Kalman filter. Taken together, the model with the Kalman filter adequately described the ventilation data and gave reliable predictions of the probability of apnea.

### Simulations.

**Figure 8:**
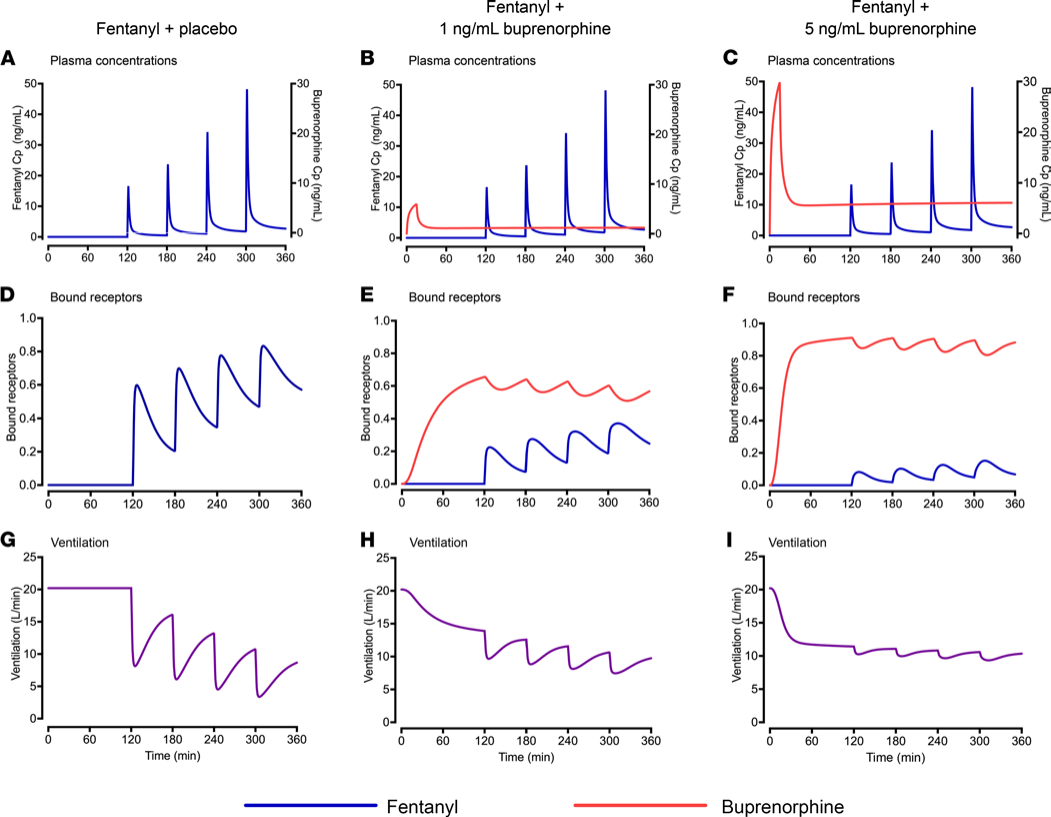
Results of simulation study: probabilities of apnea and decrease in ventilation. Simulations in a representative (“typical”) individual with chronic opioid use showing the effect of 4 subsequent fentanyl i.v. doses (0.25, 0.35, 0.50, and 0.70 mg/70 kg) on top of a buprenorphine plasma concentration of 0 (placebo), 1, and 5 ng/mL. (**A**–**C**) Fentanyl and buprenorphine plasma concentrations (Cp). (**D**–**F**) Fentanyl and buprenorphine receptor occupancy. (**G–I**) Ventilation.

**Figure 9:**
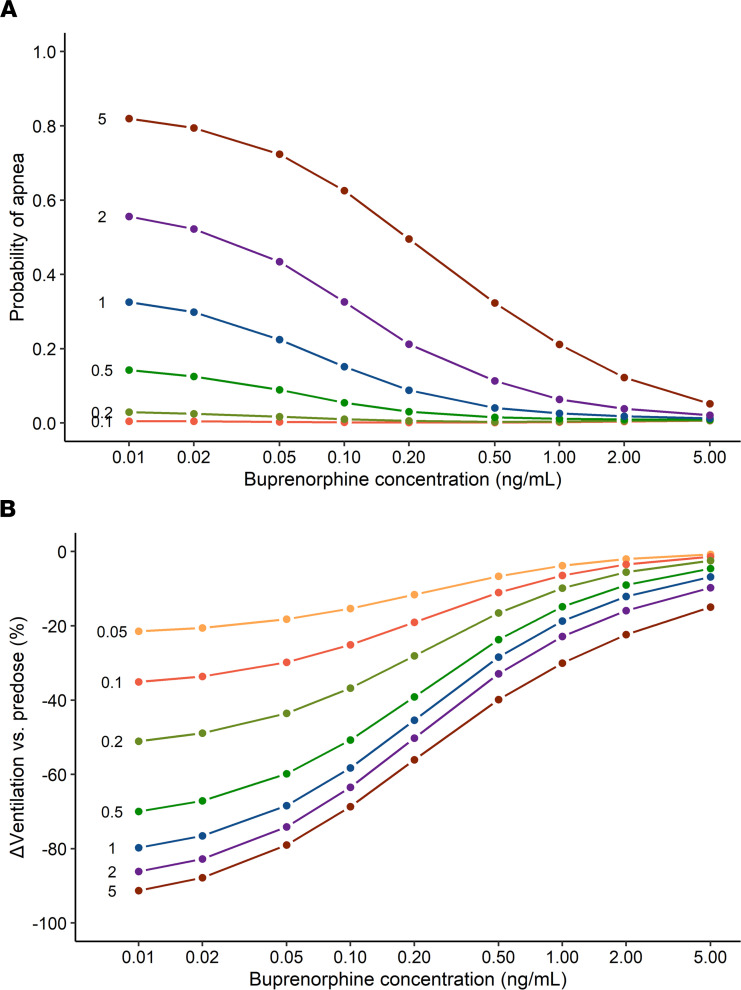
Results of simulation study: plasma concentrations, receptor binding, and ventilation. Simulations showing the probability of apnea (**A**) and median peak decrease in ventilation (**B**) at various fentanyl bolus doses ranging from 0.05 to 5 mg/70 kg (dose in mg/70 kg given within the panels before their respective effect lines) against the steady-state buprenorphine plasma concentration in individuals with chronic opioid use. The peak drop in ventilation was calculated from pre-fentanyl value and expressed a percentage of ventilation baseline (V_B_).

Simulations were performed to better understand the interaction between fentanyl and buprenorphine on ventilation in individuals with chronic opioid use. In Figure 8, we show the effect of 4 subsequent i.v. fentanyl doses, 0.25, 0.35, 0.50, and 0.70 mg/70 kg, at 3 target buprenorphine plasma concentrations, 0 (placebo), 1, and 5 ng/mL. Figure 8, D–F, show that buprenorphine reduced the number of fentanyl-bound receptors in a concentration-dependent manner, resulting in a smaller fentanyl effect on ventilation (Figure 8, G–I) (see Supplemental Figure 7 for complete results, including 2 ng/mL buprenorphine). Figure 9A shows the probability of fentanyl-induced apnea as a function of buprenorphine plasma concentration at increasing doses of fentanyl (0.1 to 5 mg/70 kg); the probability was calculated as the percentage of simulated individuals experiencing at least 1 episode of apnea. Overall, simulation results aligned with experimental results in the study. During the placebo session, 2 of 8 (25%) individuals with chronic opioid use had apnea with a cumulative fentanyl dose of 0.6 mg/70 kg, and 7 of 8 (88%) had apnea after a cumulative fentanyl dose of 1.8 mg/70 kg. Under placebo conditions, the predicted percentage of study participants with apnea was 16% for 0.5 mg/70 kg fentanyl and 59% for 2 mg/70 kg fentanyl. During the buprenorphine session, fentanyl effects on ventilation were reduced, and only 1 of 8 (13%) individuals with chronic opioid use experienced apnea after a cumulative dose of 1.8 mg/70 kg. In the simulations, the percentage of individuals with apnea after 2 mg/70 kg fentanyl ranged between 2.1% (5 ng/mL buprenorphine) and 6.4% (1 ng/mL buprenorphine). Figure 9B shows the effect of increasing doses of fentanyl (0.05 to 5 mg/70 kg) on the change in minute ventilation relative to pre-fentanyl baseline as a function of buprenorphine plasma concentration. Both simulations showed that relatively high plasma concentrations of buprenorphine (2 ng/mL or higher) reduced the probability of apnea corresponding with a smaller ventilatory depression. The best clinical outcomes were observed at a buprenorphine concentration of 5 ng/mL for the highest dose of fentanyl investigated.

## Discussion

We tested whether the MOR partial agonist buprenorphine was able to effectively prevent respiratory depression induced by the MOR full agonist fentanyl in opioid-naive volunteers and in individuals with chronic opioid use. The relationship between opioid (fentanyl and buprenorphine) plasma concentrations and respiratory effects were analyzed using a population pharmacokinetic/pharmacodynamic modeling approach. The following are the main results from the analyses: (a) The population pharmacokinetic/pharmacodynamic model, developed from the combined analysis of data from opioid-naive volunteers and those with chronic opioid use, was able to adequately describe the complex interaction of buprenorphine and fentanyl on ventilation using receptor association/dissociation models. (b) The parameter α, which incorporates intrinsic ligand activity and receptor reserve, was 1 for fentanyl in both populations but differed between the 2 populations for buprenorphine, with values of 0.8 in opioid-naive volunteers and just 0.5 in individuals with chronic opioid use; the lower α for buprenorphine is consistent with the previously reported buprenorphine ceiling effect on respiratory depression (8). (c) Fentanyl sensitivity differed 3.3-fold between the 2 populations, with reduced fentanyl sensitivity in individuals with chronic opioid use (C_50,F_ = 2.23 ng/mL) compared with opioid-naive individuals (C_50,F_ = 0.68 ng/mL), indicative of substantial tolerance to the respiratory effects of fentanyl in individuals with chronic opioid use. (d) Despite the decreased fentanyl sensitivity in individuals with chronic opioid use, apnea did occur in this population with a probability approaching 1 at high-dose fentanyl administration (Figure 9); consequently, a protective pharmacological approach remains a necessity in this particular population. (e) Buprenorphine displayed slow receptor kinetics with a dissociation rate constant k_OFF_ estimated at 0.019 min^–1^, which corresponds to a half-life of 37 minutes. (f) Like fentanyl, buprenorphine sensitivity was reduced in individuals with chronic opioid use (apparent potency K = k_OFF_/k_ON_ = 0.22 ng/mL) compared with opioid-naive individuals (K = 0.072 ng/mL). (g) The plasma/effect-site equilibration half-life, t_½__ke0_, was 6.9 minutes for fentanyl and 251 minutes for buprenorphine; no differences between the 2 populations were noted. (h) Finally, buprenorphine produced a concentration-dependent (i.e., receptor binding dependent) reduction of the ability of fentanyl to induce respiratory depression; this was further confirmed in simulation studies showing that buprenorphine plasma concentrations of 2 ng/mL and higher exerted effective protective effects even with high-dose fentanyl administrations (e.g., 2–5 mg/70 kg).

Hence, our data demonstrated that buprenorphine formulations that deliver sustained plasma concentrations of 2 ng/mL or higher would have protective effects against respiratory depression and apnea induced by full-opioid agonists, such as fentanyl. Although medium-dose buprenorphine (target plasma concentration: 2 ng/mL) was effective toward low doses of fentanyl, high-dose buprenorphine (target plasma concentration: 5 ng/mL) appeared to provide a larger protective effect toward high doses of fentanyl, such as the 5 mg/70 kg dose as presented in the simulations.

Concentration-dependent effects resulting from the competitive interaction of buprenorphine and (ab)used opioid at the MOR are also noted for other clinical effects of buprenorphine during treatment of opioid use disorder. In a study evaluating the ability of a buprenorphine extended-release formulation (BUP-XR) to block the subjective effects of the full MOR agonist hydromorphone, buprenorphine plasma concentrations of 2 ng/mL or higher produced a more consistent response toward opioid blockade (11). Additionally, a subgroup analysis of a phase III randomized clinical trial of BUP-XR indicated that patients who used opioids by the i.v. route (thereby exposed to higher concentrations of opioids) had higher abstinence rates following plasma exposure to 5–6 ng/mL buprenorphine (maintenance dose of 300 mg) compared with 2–3 ng/mL buprenorphine (maintenance dose of 100 mg) (12).

The mechanisms of the reduction in opioid sensitivity in individuals with chronic opioid use relative to opioid-naive individuals, as described by differences in fentanyl and buprenorphine potency parameters C_50_ and K (k_OFF_/k_ON_), respectively, have been discussed previously (13). In brief, apart from possible pharmacokinetic effects, such as upregulation of efflux transporters in brain endothelial cells, the observation of a reduced opioid potency in individuals with chronic opioid use is related to one of several cellular and molecular changes that occur during chronic opioid exposure, including receptor desensitization, endocytosis, degradation, and downregulation. Possible mediators in these processes are PKC, β-arrestin, adenylate cyclase, NMDA receptors, and glial cells (13). All of these factors are involved in the development of tolerance to opioid analgesia; only PKC has been shown to be involved in the tolerance to opioid-induced respiratory depression (14).

The effects of fentanyl and buprenorphine on the ventilatory control system have been previously described in opioid-naive volunteers, albeit never in combination and not in individuals with chronic opioid use (7–9, 15). The parameter estimates of the current study obtained in opioid-naive individuals are in close agreement with these prior findings. We previously observed that the ability of the competitive MOR antagonist naloxone to reverse buprenorphine-induced respiratory depression is reduced compared with reversal of fentanyl- and morphine-induced respiratory depression (7, 8, 16). We related this to the slow buprenorphine receptor kinetics and related high receptor affinity, and these same factors contribute to our current findings that buprenorphine had protective effects against fentanyl-induced respiratory depression. When buprenorphine receptor occupancy is sufficiently high, fentanyl with its rapid receptor kinetics is unable to activate the MOR and consequently will not cause additional respiratory depression on top of the respiratory effects of buprenorphine. Buprenorphine respiratory effects are imposed by its intrinsic activity, which was relatively small in individuals with chronic opioid use. Although the study was conducted in a controlled setting in a relatively small number of individuals with chronic opioid use and may not be broadly applicable to the range of patients treated for opioid use disorder, this is an important finding and supports the protective effect of buprenorphine in limiting life-threatening respiratory depression in individuals with chronic opioid use, at a background of just asymptomatic respiratory depression caused by buprenorphine alone. These data warrant further investigation in a large outcome study where a BUP-XR formulation maintaining plasma concentrations at or above 2 ng/mL could be used.

To accurately describe the variability in the data and improve the accuracy of model parameter estimates, we incorporated a Kalman filter. Compared with the pharmacokinetic/pharmacodynamic model without a Kalman filter, adding a noise filter reduced the presence of autocorrelations and cross-correlations within the model residuals and resulted in improved model predictions. We discussed previously that this indicates improvement in model performance with more reliable estimates of variability and structural model parameters (17). If we compare the model parameter estimates of the final models with and without a Kalman filter, the differences are relatively small. Most importantly, the speed of onset/offset of the fentanyl response increased from 18.7 to 6.9 minutes (t_½ ke0_), whereas the buprenorphine dissociation rate constant k_OFF_ remained similar (t_½__kOFF_ = 43 versus 37 minutes). The fentanyl t_½__ke0_ of 6.9 minutes is a more realistic estimate of fentanyl dynamics as it corresponds with its value for changes in the power spectrum of the electroencephalogram (t_½__ke0_ = 6.4 minutes) (18). Earlier analyses of pharmacokinetic and pharmacokinetic/pharmacodynamic data sets similarly favored stochastic models with a Kalman filter to remove correlated residual noise (17, 19, 20).

### Conclusions.

Buprenorphine has been shown to reduce all-cause mortality and opioid-related mortality following treatment after a nonfatal opioid overdose (4, 21). Although it is well established that maintenance treatment with buprenorphine reduces illicit opioid use, the current study describes a second mechanism through which buprenorphine may reduce opioid overdose deaths. Our data showed that buprenorphine had a protective effect against fentanyl-induced respiratory depression at plasma concentrations of 2 ng/mL and higher, with a reduced probability of apnea even at high fentanyl doses. This indicates that when buprenorphine receptor occupancy is sufficiently high, fentanyl is unable to activate the MOR and consequently will not cause additional respiratory depression on top of the mild respiratory effects of buprenorphine. Although this small experimental medicine study was not performed in real-life conditions and warrants the conduct of a large outcome study, the ability of buprenorphine to reduce the risk of serious respiratory events was clearly demonstrated.

## Methods

The study was conducted from March 2018 through January 2019 in Leiden, Netherlands. Dosing and respiratory testing were performed at the Department of Anesthesiology of the Leiden University Medical Center; all other procedures were performed at the Centre for Human Drug Research, both located in Leiden, Netherlands. The study design and primary clinical outcomes were previously published (10); the modeling of fentanyl versus placebo responses were previously published (13). Here, we report on the population pharmacokinetic/pharmacodynamic modeling of the complete data set characterizing the buprenorphine-fentanyl interaction in opioid-naive volunteers and those with chronic opioid use.

### Study design

The study had 2 parts: part A was conducted in opioid-naive volunteers and part B was conducted in individuals with chronic opioid use treated for chronic pain or opioid use disorder (see CONSORT flow diagram in Figure 1).

#### Part A.

Part A had a single-blind, 2-sequence crossover design and was performed in 14 nonsmoking (including e-cigarettes) healthy study participants of either sex, aged 18–45 years, with a BMI of 18–30 kg/m^2^ and without any history of any medical or psychiatric disease, including substance use disorder. Study participants were randomized to receive a continuous i.v. infusion of buprenorphine or a placebo on 2 occasions (period 1 and period 2). The randomization schedule was generated by an independent statistician using PC SAS version 9.4. Up to 4 identical i.v. escalating fentanyl challenges (in mg/70 kg) were administered in both periods on top of a buprenorphine or placebo infusion (Table 1). In some study participants, a third period (period 3) was added, in which they only received a buprenorphine infusion in an open-label manner. Participation in period 3 was optional. The time between study periods was 10–17 days for adequate washout.

#### Part B.

Part B had an open-label, single-sequence crossover design and was conducted in 8 opioid-tolerant individuals, aged 18–55 years, with a BMI of 18–32 kg/m^2^ and using at least 90 mg oral morphine equivalents daily. All participants were only enrolled if they were in stable physical and mental condition as defined by the investigators and based on their medical, neurological, and psychiatric history; blood and urine chemistry; and electrocardiogram. They were admitted to the clinic 2–5 days prior to period 1 and, if not already using oxycodone, transitioned to oral oxycodone. To ensure washout of each participant’s usual opioids, tailored substitution schedules with oxycodone began a minimum of 48 hours before the first experiment, and the last dose of oxycodone was administered at least 15 hours before any study drug administration. During period 1, all participants received 4 escalating fentanyl i.v. doses on top of a placebo infusion. During period 2, the participants received a buprenorphine infusion combined with the identical fentanyl doses (in mg/kg) as given during period 1. This fixed dosing sequence was chosen to optimize the fentanyl dose escalation before the fentanyl-buprenorphine combination was administered. Because of the short half-life of fentanyl, period 2 occurred at least 40 hours after period 1. During this washout period, participants again received oxycodone for opioid substitution.

On the experiment days, all study participants were transferred to an anesthesia suite where they received an i.v. line in one arm for drug administration and an arterial line in the contralateral radial artery for blood sampling. Arterial oxygen saturation was measured by finger probe (Masimo Corporation) and heart rate using 3 chest electrodes (Datex Cardiocap).

### Respiratory testing

Ventilation was measured on a breath-to-breath basis at iso-hypercapnia and iso-normoxia, using the end-tidal forcing technique (22, 23). The end-tidal oxygen concentration was kept constant at 13.5 vol.%, and the end-tidal CO_2_ concentration was kept constant at the level that caused a minute ventilation of at least 20 L/min at baseline (i.e., prior to any drug administration). See previous studies (22, 23) for a detailed description of the technique. In brief, a face mask was placed over the nose and mouth, which was connected to a pneumotachograph/pressure transducer system (Hans Rudolph Inc.) for measurement of ventilation and 3 mass flow controllers (Bronkhorst High Tech) maintained delivery of O_2_, CO_2_, and N_2_. The mass flow controllers were driven by a computer running custom-made software RESREG/ACQ (Leiden University Medical Center, Netherlands) allowing strict control of inspired gas concentrations. Gas concentrations were measured at the mouth by a capnograph (Datex Capnomac). Respiration was measured from placement of the mask until the end of the buprenorphine or placebo infusion. All breath-to-breath data were averaged over 1 minute for pharmacokinetic/pharmacodynamic data analyses.

### Drug dosing in part A

After ventilation had stabilized, a 6-hour continuous infusion of buprenorphine (Indivior UK Ltd.) or placebo (normal saline) was started. Two buprenorphine dose cohorts, low and high, were evaluated, targeting 7 individuals per cohort and aiming to achieve buprenorphine plasma concentrations of 0.2 and 0.5 ng/mL. Escalating i.v. bolus doses of fentanyl (Hameln Pharmaceuticals Ltd.) were administered in the range of 0.075–0.35 mg/70 kg at t = 2, 3, 4, and 5 hours after the start of the buprenorphine or placebo infusion. See Table 1 for the buprenorphine and fentanyl dosing schemes. Fentanyl dose escalations were limited if a procedure-related adverse event occurred, defined by the loss of respiratory activity for 60 seconds or longer despite active stimulation of the participant, end-tidal CO_2_ concentration greater than 67.5 mmHg, oxygen saturation less than 85% for at least 2 minutes, or any other situation or condition that could interfere with the health of the participant as judged by the investigators. If an apneic event occurred and the individual was verbally stimulated to breathe, the individual did not proceed to the next fentanyl dose level.

### Drug dosing in part B

After ventilation had stabilized, a 6-hour continuous infusion of buprenorphine or placebo was started. Three buprenorphine dose cohorts, low, medium, and high, were evaluated with 2–3 individuals per group aiming to achieve buprenorphine plasma concentrations of 1, 2, and 5 ng/mL, respectively, corresponding to 50%, 70%, and 80% MOR occupancy (24). Escalating i.v. bolus doses of fentanyl were administered in the range of 0.25–0.70 mg/70 kg at t = 2, 3, 4, and 5 hours after the start of the buprenorphine or placebo infusion. See Table 1 for the buprenorphine and fentanyl dosing schemes. As for opioid-naive volunteers, fentanyl dose-escalation was limited when a procedure-related adverse event occurred.

### Adjudication of respiratory data

The minute ventilation data collected in part A and part B were adjudicated to account for the impact of concurrent events, such as stimulation to breathe, face mask removal, urinating while the face mask was on, and severe itching. The adjudication of the data, driven by clinical observations and inspection of the raw data, was performed as follows: (a) minute ventilation data measured under respiratory stimulation during a period of respiratory arrest were set to zero (apnea), indicating that there was no spontaneous breathing; (b) minute ventilation data measured within ± 5 minutes of the mask removal were set to missing due to artefacts in ventilation associated with the removal/placement of the mask; (c) minute ventilation data measured while the study participant was urinating while in the face mask were set to missing values over the corresponding time interval ± 5 minutes; (d) minute ventilation data measured while the study participant experienced severe itching leading to the elevation of ventilation were set to missing values over the corresponding time interval ± 5 minutes; and (e) for apnea events lasting less than 60 seconds, minute ventilation was corrected for zero values during apnea (weighted average). Data adjudication was performed in R software version 3.5.1.

### Blood samples and fentanyl and buprenorphine assays

In parts A and B, 8 mL arterial blood samples were drawn at predefined times as specified in Table 1 for measurement of fentanyl and buprenorphine plasma concentrations. When no fentanyl bolus was given, sampling continued at hourly intervals and again according to schedule from 360 minutes on. Plasma was separated within 30 minutes of blood collection and stored at −20°C until analysis. Plasma concentrations of buprenorphine and fentanyl were determined using 2 validated liquid chromatography with tandem mass spectrometry (LC-MS/MS) assays. Briefly, human K_2_EDTA plasma containing the analytes and the deuterated internal standards (buprenorphine-d4 or fentanyl-d5) were extracted with methyl tert-butyl ether/hexane for buprenorphine or with methyl tert-butyl ether after the addition of sodium carbonate for fentanyl (liquid-liquid extraction). After extraction, the organic phase was dried down under nitrogen in a water bath at 40°C, reconstituted, and transferred to plastic injection vials for buprenorphine. For fentanyl, a small portion of the organic phase obtained after extraction was transferred to an autosampler vial that contained formic acid in water. The peak area of the *m/z* 468.5→414.4 buprenorphine product ion was measured against the peak area of the *m/z* 472.5→414.4 buprenorphine-d4 internal standard production. The peak area of the *m/z* 337→188 fentanyl product ion was measured against the peak area of the *m/z* 342→188 fentanyl-d5 internal standard product ion. Quantitation was performed using weighted (1/x^2^) linear least squares regression analyses generated from calibration standards. Both assays were fully validated for linearity, selectivity, recovery, matrix effect, accuracy, precision, and stability before application to the sample analysis. The calibration range was 0.020–10.0 ng/mL for buprenorphine and 0.100–50.0 ng/mL for fentanyl. The overall accuracy and precision for quality control samples during the sample analyses were all within 5.3%. All plasma samples were analyzed within the established stability window.

### Statistics

#### Pharmacokinetic/pharmacodynamic analyses.

The analyses were conducted in 2 stages (sequential pharmacokinetic/pharmacodynamic modeling). In a first stage, population pharmacokinetic models were fitted to buprenorphine and fentanyl plasma concentration data. In a second stage, empirical Bayes estimates of individual pharmacokinetic parameters obtained from population pharmacokinetic modeling served as input for the pharmacokinetic/pharmacodynamic model describing the respiratory interaction of buprenorphine and fentanyl. Data analyses were performed using NONMEM, version 7.4.4 (ICON Development Solutions), a nonlinear mixed effects modeling software package. Perl-speaks-NONMEM (PsN; https://uupharmacometrics.github.io/PsN/) version 4.9.0 was used to operate NONMEM.

#### Pharmacokinetic model development.

Population pharmacokinetic models of buprenorphine and fentanyl were developed separately; 2- and 3-compartment models were evaluated for both drugs. Interindividual variability was estimated assuming log-normal distributions for individual pharmacokinetic parameters. When pharmacokinetic data were obtained at multiple occasions, additional random effects were included to estimate between-occasion variability. Clearances and volumes were allometrically scaled by body weight (standardized to a body weight of 70 kg), using the well-established power model and exponents of 0.75 for clearances and 1 for volumes of distribution (25). Model selection was based on standard diagnostic plots, changes in minimum objective function value, and the robustness/precision of parameter estimates. The likelihood ratio test was applied to nested models with a nominal α-level of 0.05.

Given the small sample size (22 individuals in total) and because the purpose of analysis was to provide individual predictions for pharmacokinetic/pharmacodynamic modeling, only 1 covariate was explored, i.e., the opioid use state, to evaluate differences between studied populations. Differences between populations were tested on each pharmacokinetic parameter using an automated procedure by PsN’s stepwise covariate model building utility (forward selection: *P* < 0.05; backward selection: *P* < 0.001).

#### Pharmacokinetic/pharmacodynamic model development.

Negative ventilation data were allowed by the model to describe long periods of apnea and were censored at zero using M3 methodology (26). Model estimation was performed in NONMEM using the stochastic approximation expectation-maximization (SAEM) algorithm. The importance sampling (IMP) algorithm was used to calculate the –2 log likelihood value at the final model parameter estimates and to obtain the asymptotic standard errors of estimates.

As the purpose of the present analysis was to assess the interaction of buprenorphine and fentanyl on ventilation via activation of the MOR system, receptor association/dissociation models were used and combined with biophase distribution models to account for buprenorphine and fentanyl hysteresis. The equations describing receptor association/dissociation for each molecule are as follows (8, 16):

d[BR]/dt = k_ON,B_ × [B] × [R] – k_OFF,B_ × [BR]

d[FR]/dt = k_ON,F_ × [F] × [R] – k_OFF,F_ × [FR]

where B, F, and R denote buprenorphine, fentanyl, and receptors, respectively; [B] and [F] denote effect-site concentrations for buprenorphine and fentanyl (i.e., concentrations in their respective effect compartment). [BR] and [FR] denote the concentrations of receptors bound to buprenorphine and fentanyl, respectively. [R] denotes the concentration of unbound receptors, and k_ON_ and k_OFF_ are association and disassociation rate constants, respectively. Because the dissociation rate constant for fentanyl (k_OFF,F_) was large in previous analyses (8), we assume that k_ON,F_ × [F] × [R] – k_OFF,F_ × [FR] = 0, leading to [FR] = [F] × [R]/C_50,F_ with C_50,F_ = k_OFF,F_/k_ON,F_ or the fentanyl concentration at the postulated effect-site causing 50% of maximal depression of ventilation.

Assuming that the total number of receptors [R_TOT_] is equal to the sum of drug-bound receptors and unbound receptors: [R_TOT_] = [R] + [BR] + [FR], and after normalizing [BR] and [FR] by setting [R_TOT_] = 1, we obtain: [FR] = (1 – [BR]) × ([F]/C_50,F_) / (1 + [F]/C_50,F_).

The relationship between the bound receptor concentrations and ventilation was described using a linear transduction function as follows (8):

V_E_ = V_B_ × (1 – α_B_ × [BR] – α_F_ × [FR])

where V_E_ is minute ventilation, V_B_ is the iso-hypercapnic baseline ventilation (i.e., prior to any drug given), and α_B_ and α_F_ are parameters for buprenorphine and fentanyl, respectively, that combine receptor reserve and intrinsic ligand activity.

Interindividual variability was estimated assuming log-normal distributions for individual pharmacokinetic/pharmacodynamic parameters. An additive model structure was tested for the residual error, with and without inclusion of interindividual variability. Overestimation of interindividual variability can occur when intraindividual stochastic noise processes are not appropriately accounted for (27). With the present data sets, there were 350–450 minute-ventilation measurements per individual, and those data points were most likely correlated. Hence, the standard modeling assumption that observations would be independent and normally distributed conditionally to individual-specific random effects may not be valid. Therefore, a simple Kalman filter was added to model process noise (19, 28, 29). The Kalman filter has 3 components: σ = the standard deviation of the residual (intraindividual) noise, σ_ν_ = the standard deviation of the parallel noise (which is also intraindividual), and τ the time constant determining correlation of process noise in time. Interindividual variability and interoccasion variability were tested on some of those parameters (σ and σ_ν_).

The effect of opioid-use state was first examined based on the empirical Bayes estimates of individual random effects (η) obtained from the base model and evaluated graphically and statistically by Kruskal-Wallis rank-sum test. Significant (*P* < 0.05) relationships were further tested in NONMEM using a forward/backward selection procedure. Significance levels of 0.05 and 0.001 were used for forward and backward selection, respectively. Additional criteria for covariate selection included the pharmacological relevance of the effect and the convergence of the estimation and covariance routines.

#### Model evaluation.

pvcVPCs were generated to ensure that the models were able to reproduce the data used for model building. Additionally, standard goodness-of-fit plots were produced, including individual and population diagnostic plots. When the Kalman filter was implemented, autocorrelation (i.e., correlation between residuals shifted by Δt) functions and cross-correlation functions (correlation between residuals and pharmacodynamic model input shifted by Δt) were plotted and inspected for model inadequacies according to Ljung (28). When the residuals are white (uncorrelated), the autocorrelation function is zero if Δt > 0; when Δt = 0, a residual has a correlation of 1 with itself. If the model fully explains the data, the cross-correlation is zero (i.e., the residuals are completely random).

### Data availability

All relevant study data are located at Dryad: https://doi.org/10.5061/dryad.j3tx95xdb

### Study approval

The protocol was approved by the Medical Review and Ethics Committee of the BEBO foundation (Assen, Netherlands) and was registered at the Dutch Cochrane Centre (https://www.trialregister.nl) under identifier 7028. All participants gave written informed consent prior to the start of any study procedures, and all procedures were conducted according to EU good clinical practice guidelines and the Declaration of Helsinki.

## Author contributions

AD, CML, EO, and RLD wrote the manuscript, which was reviewed by all authors. AD, CML, EO, GJG, and RLD designed the research (study conduct, modeling); AD, GJG, LM, MHA, MN, and MVV conducted the experimental phase; AD, CML, and EO analyzed the data. EO and MHA are co–first authors. Since the results of the modeling analyses were pivotal in this work, EO is cited first.

## Supplementary Material

Supplemental data

ICMJE disclosure forms

## Figures and Tables

**Table 1:**
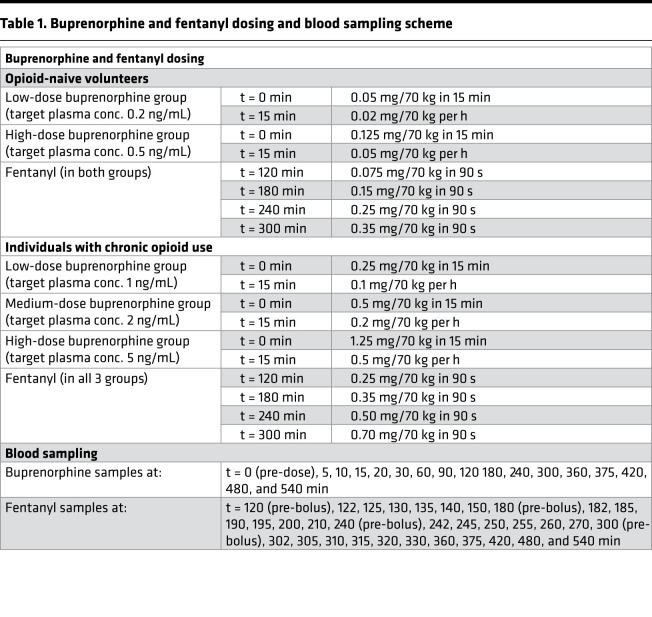
Buprenorphine and fentanyl dosing and blood sampling scheme

**Table 2:**
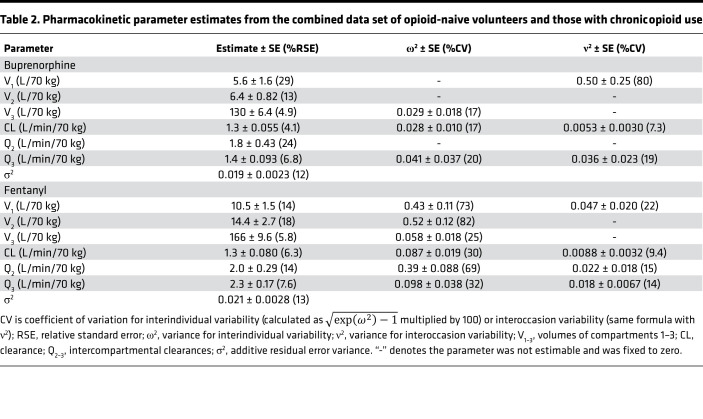
Pharmacokinetic parameter estimates from the combined data set of opioid-naive volunteers and those with chronic opioid use

**Table 3:**
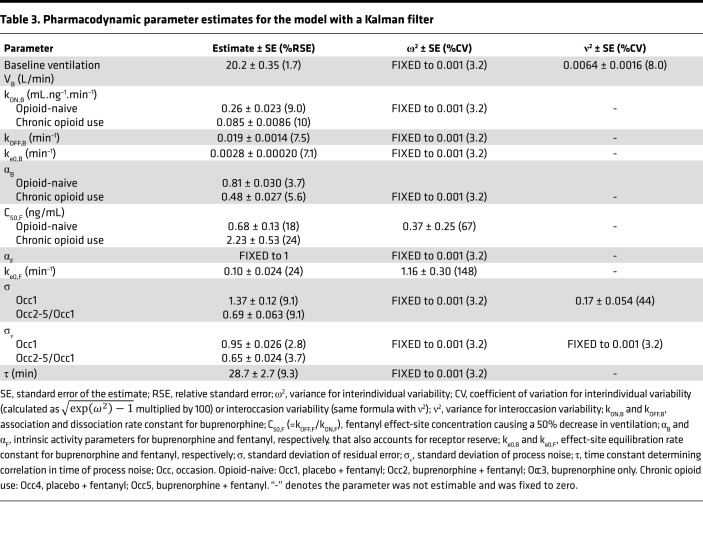
Pharmacodynamic parameter estimates for the model with a Kalman filter

## Version 1 03/22/2022

In-Press Preview

## Version 2 05/09/2022

Electronic publication
