# Supplementary material for: Modeling buprenorphine reduction of fentanyl-induced respiratory depression
Source: JCI Insight. 2022 May 9;7(9):e156973. doi: 10.1172/jci.insight.156973 (PMC9090248; doi:10.1172/jci.insight.156973)
Supplement: Supplemental data [file jciinsight-7-156973-s214.pdf]

Modelling Buprenorphine Reduction of Fentanyl-Induced Respiratory Depression

Erik Olofsen,[1*] Marijke Hyke Algera,[1*] Laurence Moss,[1,2] Robert Dobbins,[3] Geert Jan Groeneveld,[1,2] Monique van Velzen,[1] Marieke Niesters,[1] Albert Dahan,[1] Celine M. Laffont[3]

* contributed equally

1. Department of Anesthesiology, Leiden University Medical Center, 2333 ZA Leiden, The Netherlands;
2. Centre for Human Drug Research, 2333 CL Leiden, The Netherlands;
3. Indivior Inc., North Chesterfield, VA 23235, USA.

Supplemental data

Supplemental Table 1

Supplemental Figure 1

Supplemental Figure 2

Supplemental Figure 3

Supplemental Figure 4

Supplemental Figure 5

Supplemental Figure 6

Supplemental Figure 7

Supplemental Table 1. Pharmacodynamic parameter estimates for the model without Kalman filter

Parameter	Estimate ± SE (%RSE)	ω^2 ± SE (%CV)	ν^2 ± SE (%CV)
Baseline ventilation			
V_B (L/min)			
Occ1	21.8 ± 0.69 (3.1)	FIXED to 0.001 (3.2)	0.014 ± 0.0029 (12)
Occ2/Occ1	0.95 ± 0.043 (4.5)		
Occ3/Occ1	0.72 ± 0.019 (2.6)		
Occ4/Occ1	1.02 ± 0.032 (3.1)		
Occ5/Occ1	0.99 ± 0.059 (5.9)		
$k_{ON,B}$ (mL.ng^{-1}.min^{-1})			
opioid-naïve	0.46 ± 0.034 (7.4)	FIXED to 0.001 (3.2)	-
Chronic opioid users	0.12 ± 0.011 (9.6)		
$k_{OFF,B}$ (min^{-1})	0.016 ± 0.0045 (28)	1.51 ± 0.29 (188)	-
$k_{e0,B}$ (min^{-1})	0.0024 ± 0.00050 (21)	0.82 ± 0.26 (113)	-
α_B opioid-naïve			
Chronic opioid users	0.68 ± 0.089 (13)	0.13 ± 0.074 (38)	-
	0.47 ± 0.076 (16)		
$C_{50,F}$ (ng/mL)			
opioid-naïve	0.49 ± 0.13 (26)	1.14 ± 0.30 (146)	-
Chronic opioid users	1.88 ± 0.57 (31)		
α_F	1.12 ± 0.094 (8.4)	0.14 ± 0.052 (38)	-
$k_{e0,F}$ (min^{-1})	0.037 ± 0.0064 (17)	0.62 ± 0.26 (93)	-
σ			
Occ1	3.05 ± 0.37 (12)	0.038 ± 0.030 (20)	0.071 ± 0.025 (27)
Occ2/Occ1	0.47 ± 0.073 (15)		
Occ3/Occ1	0.55 ± 0.088 (16)		
Occ4/Occ1	0.82 ± 0.19 (24)		
Occ5/Occ1	0.56 ± 0.079 (14)		

CV: coefficient of variation for interindividual variability (calculated as $\sqrt{\exp(\omega^2)-1}$ multiplied by 100) or interoccasion variability (same formula with ν^2); RSE: relative standard error; SE: standard error; ω^2: variance for interindividual variability; ν^2: variance for interoccasion variability; $k_{ON,B}$ and $k_{OFF,B}$: association and dissociation rate constant for buprenorphine; $C_{50,F}$: fentanyl effect-site concentration causing a 50% decrease in ventilation; α_B and α_F: parameters for buprenorphine and fentanyl, respectively, that combine receptor reserve and intrinsic ligand activity; $k_{e0,B}$ and $k_{e0,F}$: effect-site equilibration rate constant for buprenorphine and fentanyl, respectively; σ: standard deviation of residual error; Occ: occasion.

Opioid-naïve: Occ1: placebo + fentanyl; Occ2: buprenorphine + fentanyl; Occ3: buprenorphine only.

Chronic opioid users: Occ4: placebo + fentanyl; Occ5: buprenorphine + fentanyl.

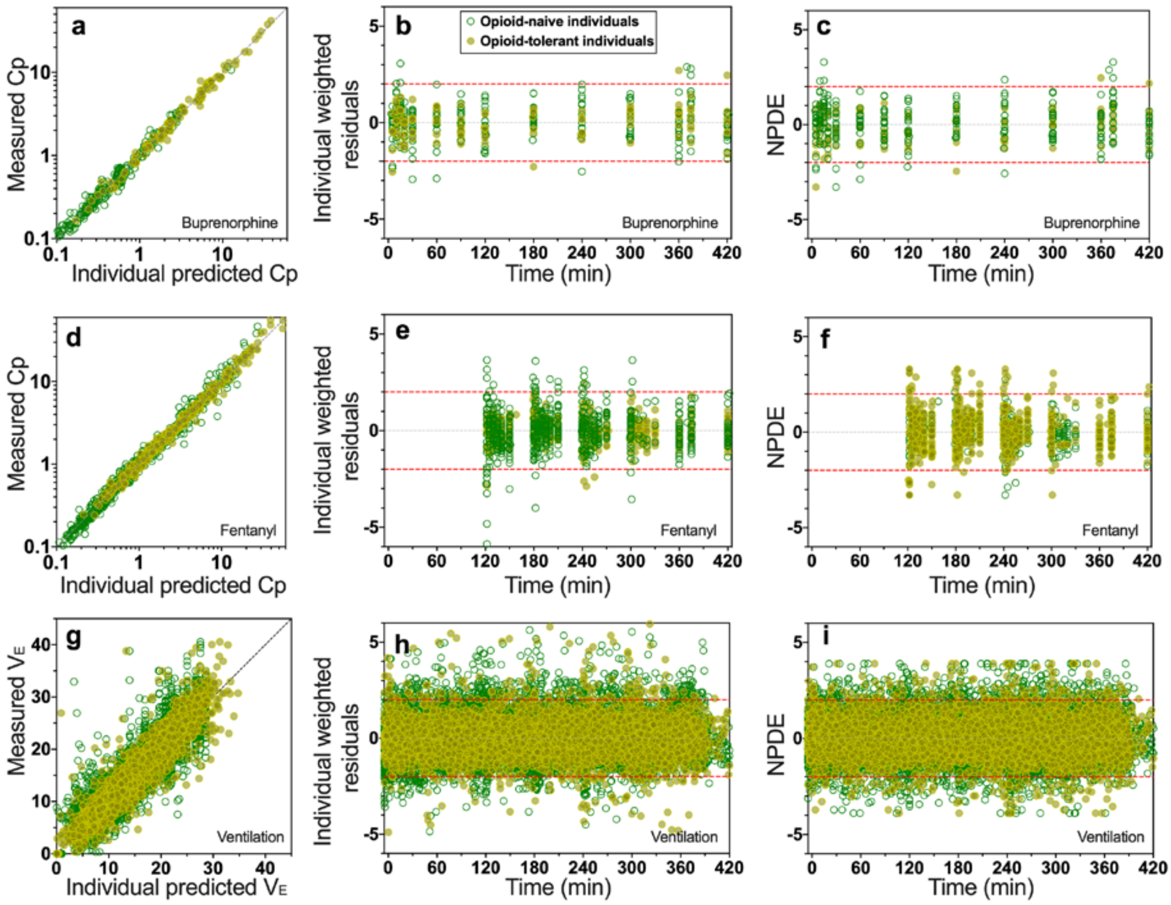

Supplemental Figure 1. Goodness-of-fits plots of the pharmacokinetic buprenorphine model (panels a, b and c), the pharmacokinetic fentanyl model (panels d, e and f), and the pharmacodynamic model (panels g, h and i). Shown are the measured values (Cp = concentration in plasma) *versus* the individual predicted values (panels a, d and g), the individual weighted residuals *versus* time (panels b, e and h) and normalized prediction distribution errors (NPDE; panels c, f and i). The red lines are ±1.96 (95% prediction interval for normal distribution).

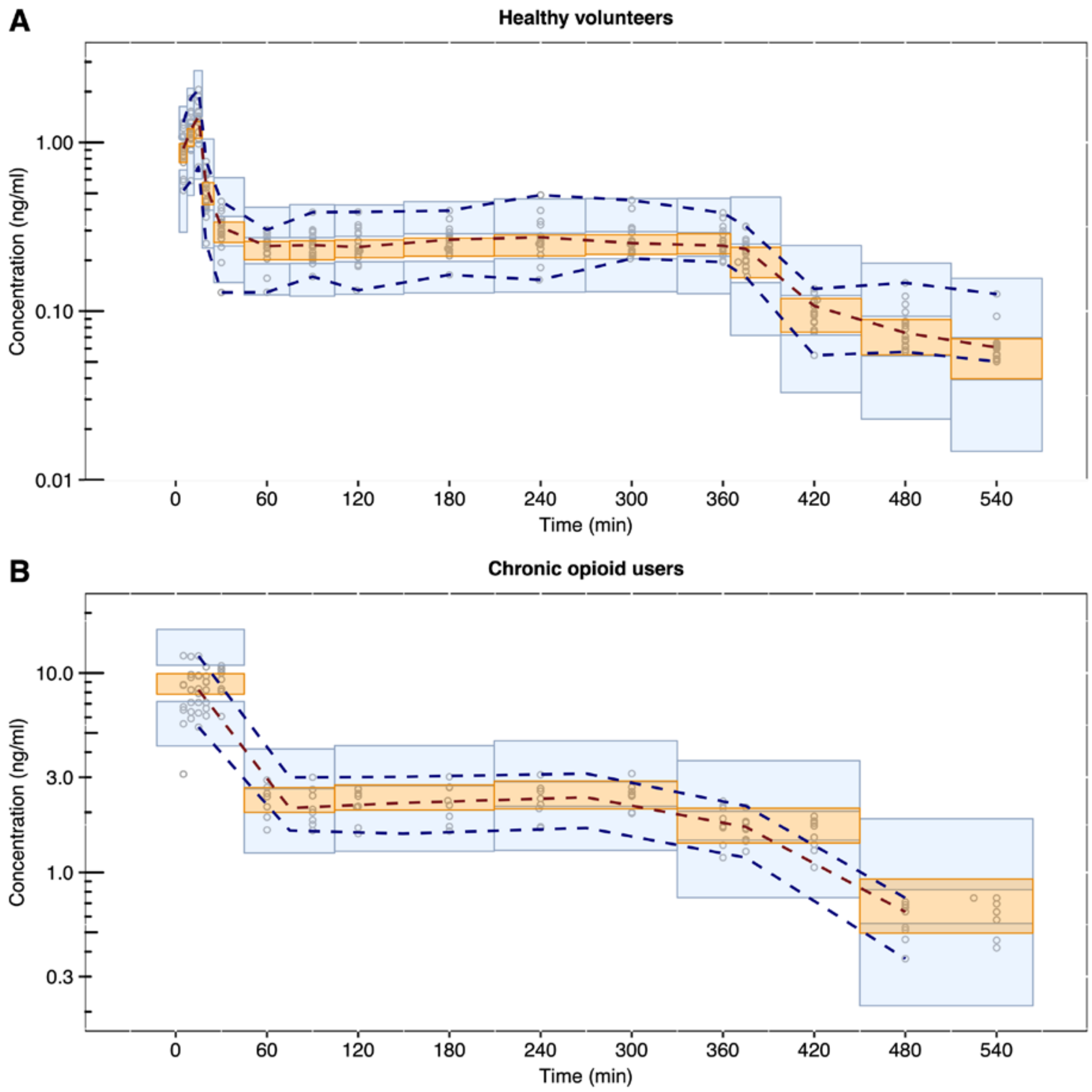

Supplemental Figure 2. Prediction- and variability-corrected visual predictive checks of the buprenorphine pharmacokinetic model in opioid-naïve volunteers (A) and chronic opioid users (B). The open circles are the measured concentrations; the broken lines are the observed percentiles (dark orange: median, dark blue: 2.5th and 97.5th percentiles); the bins are the 95% confidence intervals of simulated percentiles (orange bins: median, blue bins: 2.5th and 97.5th percentiles).

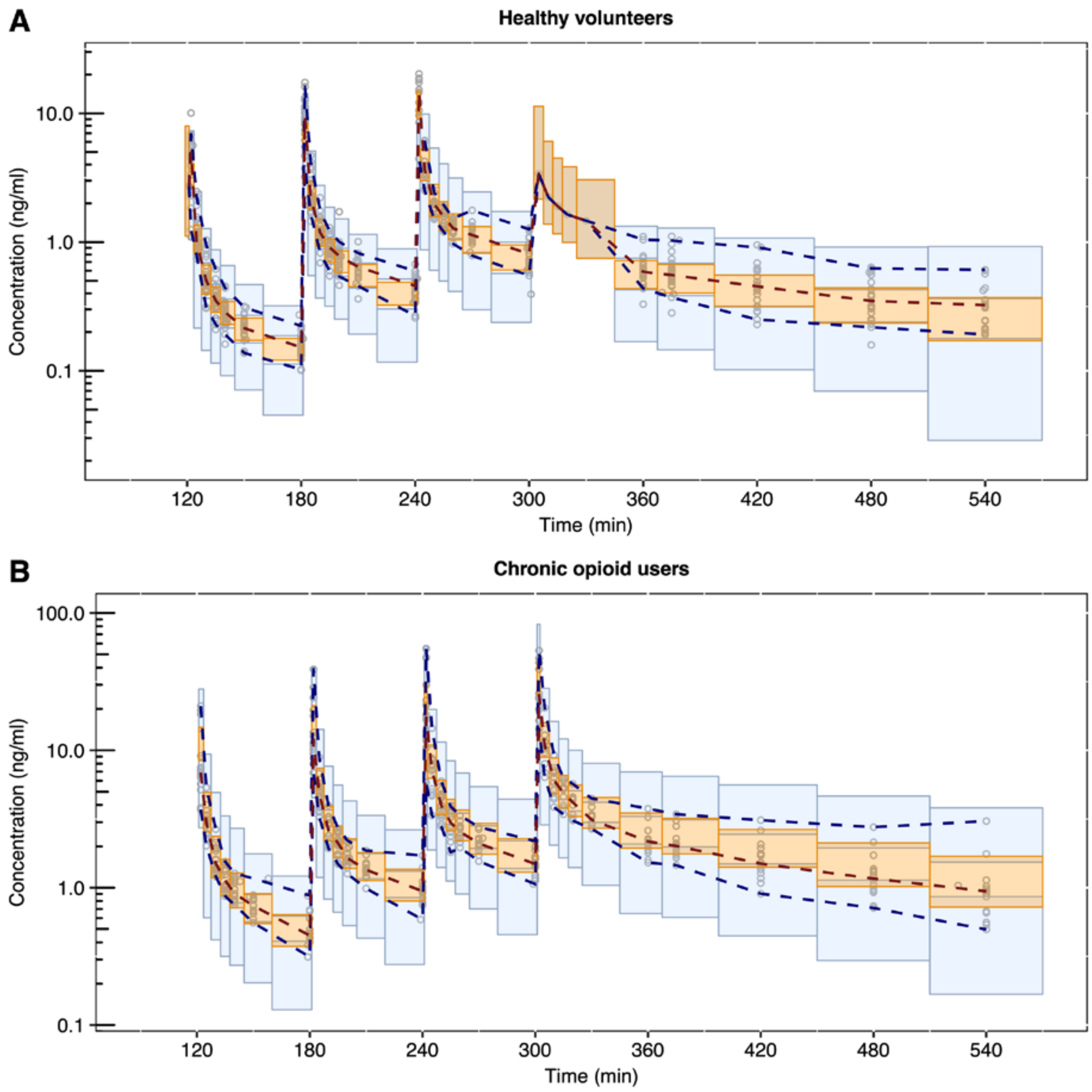

Supplemental Figure 3. Prediction- and variability-corrected visual predictive checks of the fentanyl pharmacokinetic model in opioid-naïve volunteers (A) and chronic opioid users (B). The open circles are the measured concentrations; the broken lines are the observed percentiles (dark orange: median, dark blue: 2.5th and 97.5th percentiles); the bins are the 95% confidence intervals of simulated percentiles (orange bins: median, blue bins: 2.5th and 97.5th percentiles).

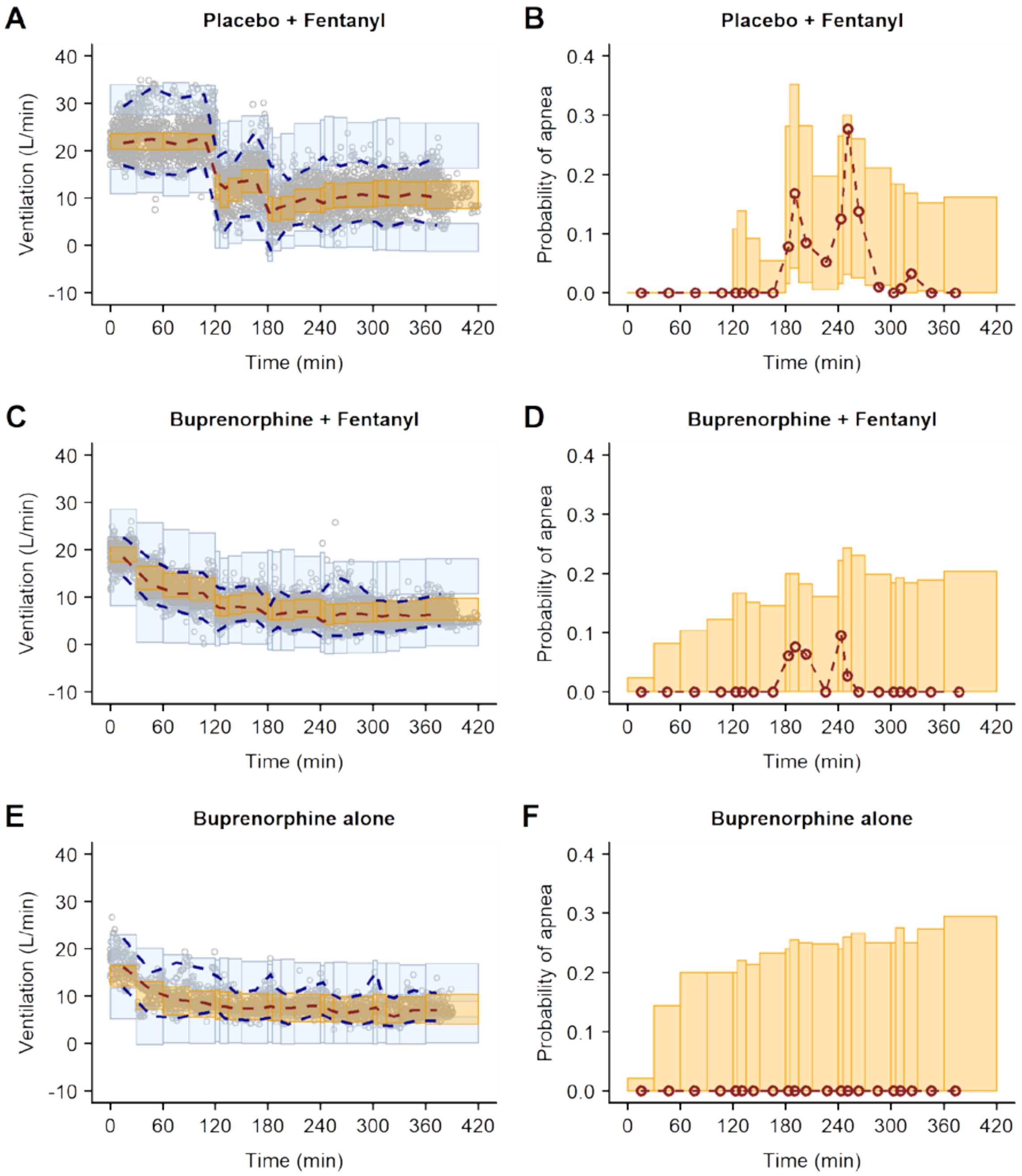

Supplemental Figure 4. Prediction- and variability-corrected visual predictive checks of the pharmacodynamic model without Kalman filter in opioid-naïve subjects for the various drug administrations and probabilities of apnea for the same conditions. A and B: Fentanyl given at the background of placebo infusion; C and D: Fentanyl given at the background of buprenorphine infusion; E and F: Just buprenorphine. The dots in panels A, C and E are the 1-min ventilation averages; the broken lines are the observed percentiles (dark orange: median, dark blue: 2.5th and 97.5th percentiles); the bins are the 95% confidence intervals of simulated percentiles (orange bins: median, blue bins: 2.5th and 97.5th percentiles). The right panels (B, D and F) give the probability of apnea. The red symbols are the probabilities of the observed apneic episodes; the orange bins are the simulated 95% confidence intervals of the probability of apnea.

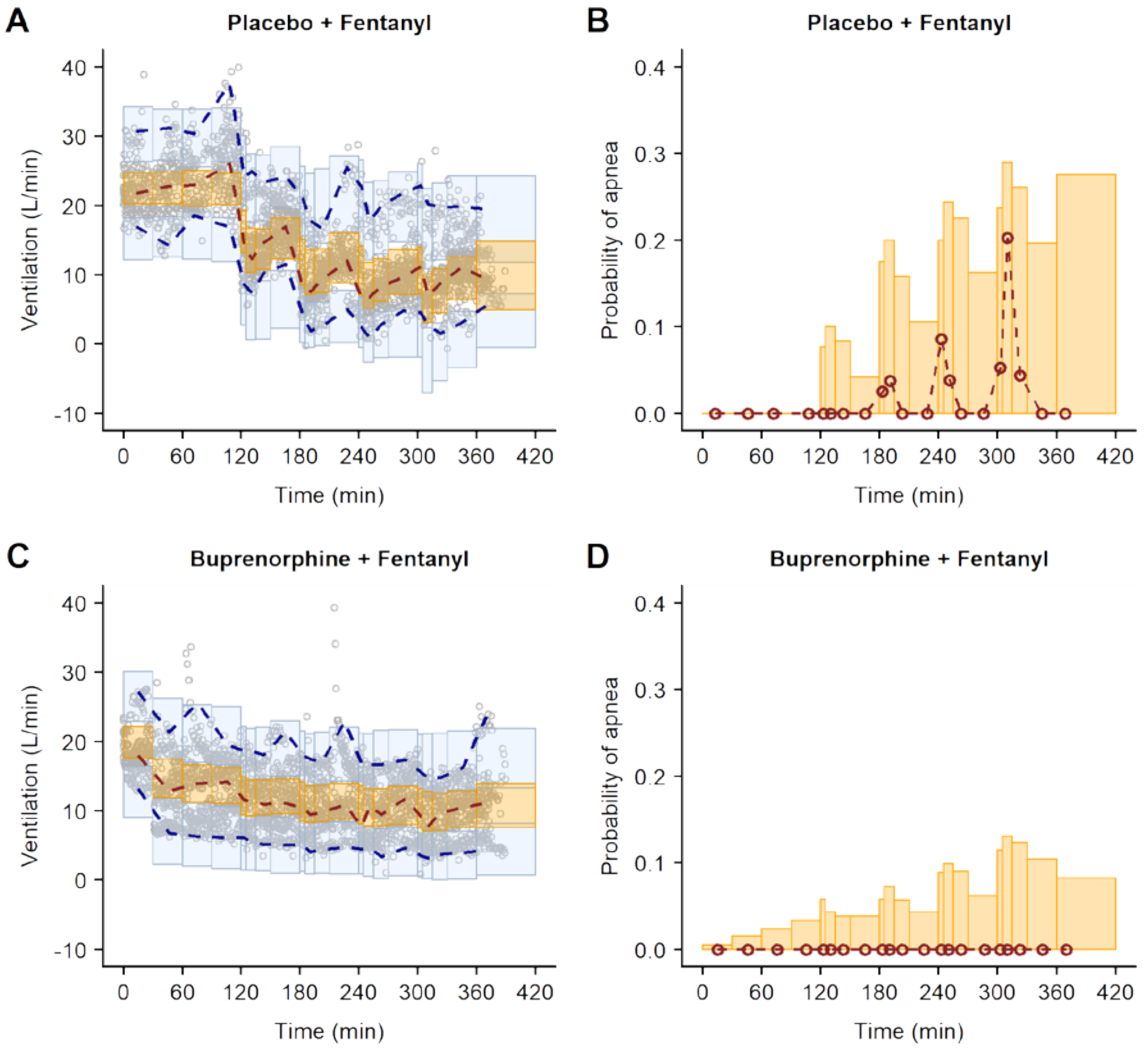

Supplemental Figure 5. Prediction- and variability-corrected visual predictive checks of the pharmacodynamic model without Kalman filter in chronic opioid users for the various drug administrations and probabilities of apnea for the same conditions. A and B: Fentanyl given at the background of placebo infusion; C and D: Fentanyl given at the background of buprenorphine infusion. The dots in panels A and C are the 1-min ventilation averages; the broken lines are the observed percentiles (dark orange: median, dark blue: 2.5th and 97.5th percentiles); the bins are the 95% confidence intervals of simulated percentiles (orange bins: median, blue bins: 2.5th and 97.5th percentiles). The right panels (B and D) give the probability of apnea. The red symbols are the probabilities of the observed apneic episodes; the orange bins are the simulated 95% confidence intervals of the probability of apnea.

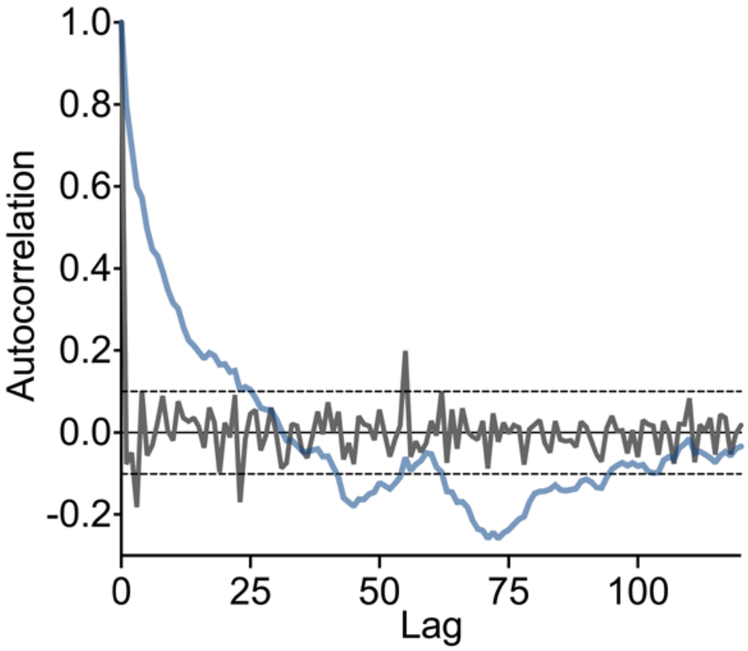

Supplemental Figure 6. An example of the autocorrelation function of the residuals of the pharmacodynamic model without Kalman filter (blue line) showing correlated noise compared to the model with Kalman filter (grey line) showing uncorrelated noise. The horizontal lines are the zero line (continuous line) ± 95% confidence intervals (broken lines).

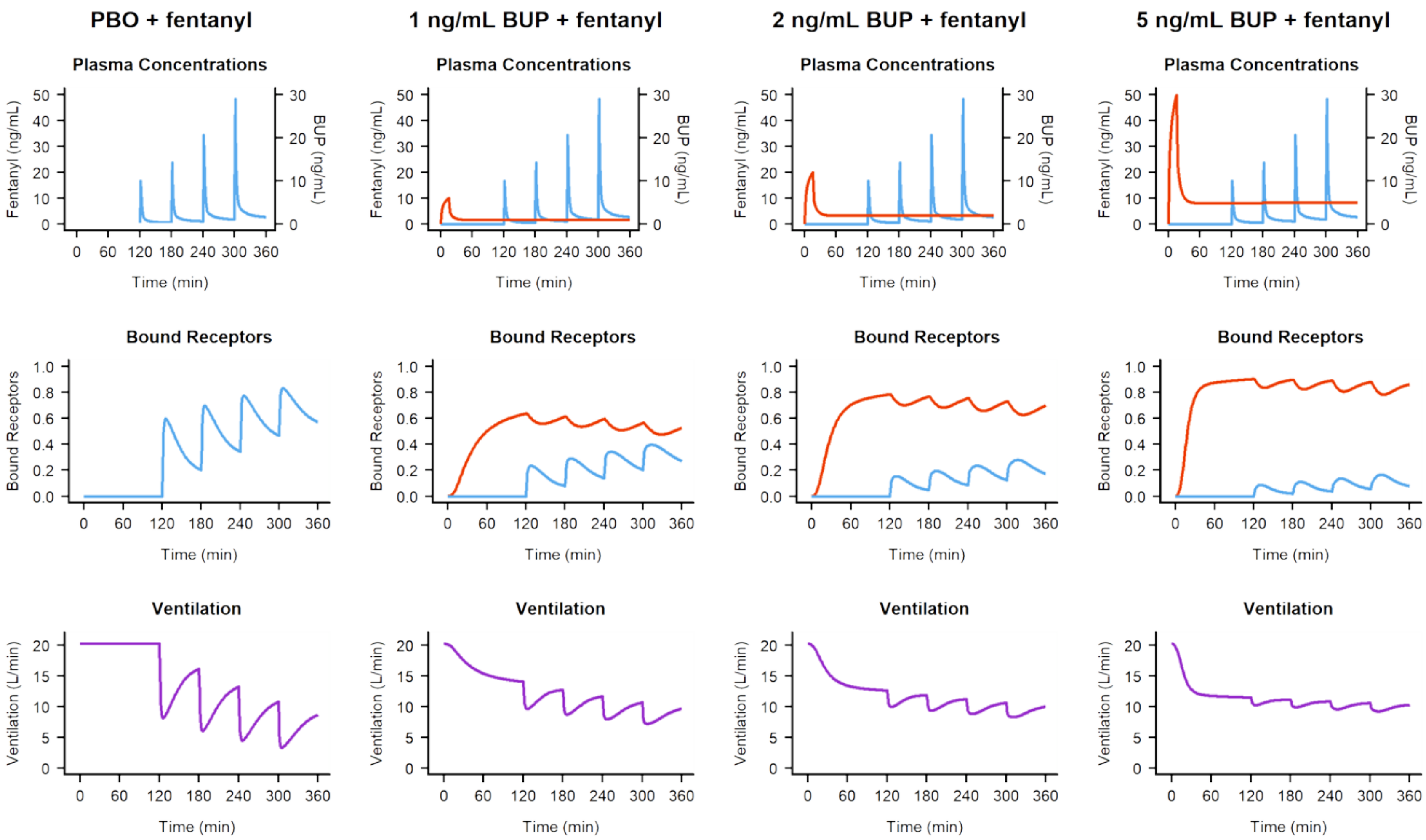

Supplemental Figure 7. Simulations in a representative ("typical") chronic opioid user showing the effect of 4 subsequent fentanyl intravenous doses (0.25, 0.35, 0.50 and 0.70 mg/70 kg) on top of a buprenorphine plasma concentration of 0 (placebo), 1, 2 and 5 ng/mL.
BUP=buprenorphine; PBO=placebo. Fentanyl predictions are shown in light blue, buprenorphine predictions in red, ventilation predictions in purple.
