# Supplementary material for: Modeling buprenorphine reduction of fentanyl-induced respiratory depression
Source: JCI Insight. 2022 May 9;7(9):e156973. doi: 10.1172/jci.insight.156973 (PMC9090248; doi:10.1172/jci.insight.156973)
Supplement: ICMJE disclosure forms [file jciinsight-7-156973-s215.pdf]

ICMJE DISCLOSURE FORM

Date: 3/4/2022

Your Name: Marijke Hyke Algera

Manuscript Title: Modelling Buprenorphine Reduction of Fentanyl-Induced Respiratory Depression

Manuscript Number (if known): 156973-INS-CMED-RV-3

In the interest of transparency, we ask you to disclose all relationships/activities/interests listed below that are related to the content of your manuscript. "Related" means any relation with for-profit or not-for-profit third parties whose interests may be affected by the content of the manuscript. Disclosure represents a commitment to transparency and does not necessarily indicate a bias. If you are in doubt about whether to list a relationship/activity/interest, it is preferable that you do so.

The author's relationships/activities/interests should be defined broadly. For example, if your manuscript pertains to the epidemiology of hypertension, you should declare all relationships with manufacturers of antihypertensive medication, even if that medication is not mentioned in the manuscript.

In item #1 below, report all support for the work reported in this manuscript without time limit. For all other items, the time frame for disclosure is the past 36 months.

		Name all entities with whom you have this relationship or indicate none (add rows as needed)	Specifications/Comments (e.g., if payments were made to you or to your institution)
**Time frame: Since the initial planning of the work**			
1	All support for the present manuscript (e.g., funding, provision of study materials, medical writing, article processing charges, etc.) **No time limit for this item.**	☐ None	
		Indivior Inc.	This study was supported by Indivior Inc., North Chesterfield, VA. The funder of the study and affiliated authors participated in designing the study, data analysis, and interpretation of the data. All authors had full access to all the data and agreed to submit the manuscript for publication.
			Click the tab key to add additional rows.
**Time frame: past 36 months**			
2	Grants or contracts from any entity (if not indicated in item #1 above).	☒ **None**	
3	Royalties or licenses	☒ **None**	

		Name all entities with whom you have this relationship or indicate none (add rows as needed)	Specifications/Comments (e.g., if payments were made to you or to your institution)
**4**	Consulting fees	☒ **None**	
**5**	Payment or honoraria for lectures, presentations, speakers bureaus, manuscript writing or educational events	☒ **None**	
**6**	Payment for expert testimony	☒ **None**	
**7**	Support for attending meetings and/or travel	☒ **None**	
**8**	Patents planned, issued or pending	☒ **None**	
**9**	Participation on a Data Safety Monitoring Board or Advisory Board	☒ **None**	
**10**	Leadership or fiduciary role in other board, society, committee or advocacy group, paid or unpaid	☒ **None**	

		Name all entities with whom you have this relationship or indicate none (add rows as needed)	Specifications/Comments (e.g., if payments were made to you or to your institution)
**11**	Stock or stock options	☒ **None**	
**12**	Receipt of equipment, materials, drugs, medical writing, gifts or other services	☒ **None**	
**13**	Other financial or non-financial interests	☒ **None**	

Please place an "X" next to the following statement to indicate your agreement:

☒ I certify that I have answered every question and have not altered the wording of any of the questions on this form.

ICMJE DISCLOSURE FORM

Date: 3/7/2022

Your Name: Albert Dahan

Manuscript Title: Modelling Buprenorphine Reduction of Fentanyl-Induced Respiratory Depression

Manuscript Number (if known): 156973-INS-CMED-RV-3

In the interest of transparency, we ask you to disclose all relationships/activities/interests listed below that are related to the content of your manuscript. "Related" means any relation with for-profit or not-for-profit third parties whose interests may be affected by the content of the manuscript. Disclosure represents a commitment to transparency and does not necessarily indicate a bias. If you are in doubt about whether to list a relationship/activity/interest, it is preferable that you do so.

The author's relationships/activities/interests should be defined broadly. For example, if your manuscript pertains to the epidemiology of hypertension, you should declare all relationships with manufacturers of antihypertensive medication, even if that medication is not mentioned in the manuscript.

In item #1 below, report all support for the work reported in this manuscript without time limit. For all other items, the time frame for disclosure is the past 36 months.

		Name all entities with whom you have this relationship or indicate none (add rows as needed)	Specifications/Comments (e.g., if payments were made to you or to your institution)
**Time frame: Since the initial planning of the work**			
1	All support for the present manuscript (e.g., funding, provision of study materials, medical writing, article processing charges, etc.)<br>No time limit for this item.	☐ None	
		Indivior Inc.	This study was supported by Indivior Inc., North Chesterfield, VA. The funder of the study and affiliated authors participated in designing the study, data analysis, and interpretation of the data. All authors had full access to all the data and agreed to submit the manuscript for publication.
			Click the tab key to add additional rows.
**Time frame: past 36 months**			
2	Grants or contracts from any entity (if not indicated in item #1 above).	☐ **None**	
		ZonMW (The Hague, The Netherlands) in the framework of the NWA-ORC Call (NWA.1160.18.300) and the US Food and Drug Administration (Washington DC, USA).	Author received grants/awards.

		Name all entities with whom you have this relationship or indicate none (add rows as needed)	Specifications/Comments (e.g., if payments were made to you or to your institution)
**3**	Royalties or licenses	☒ **None**	
**4**	Consulting fees	☐ **None**	
		Enalare Therapeutics Inc. (USA), Grünenthal BV (The Netherlands), Trevena Inc. (USA), and MSD Nederland BV.	Author received consultancy and/or speaker fees.
**5**	Payment or honoraria for lectures, presentations, speakers bureaus, manuscript writing or educational events	☐ **None**	
		Enalare Therapeutics Inc. (USA), Grünenthal BV (The Netherlands), Trevena Inc. (USA), and MSD Nederland BV.	Author received consultancy and/or speaker fees.
**6**	Payment for expert testimony	☒ **None**	
**7**	Support for attending meetings and/or travel	☒ **None**	
**8**	Patents planned, issued or pending	☒ **None**	
**9**	Participation on a Data Safety Monitoring Board or Advisory Board	☒ **None**	

		Name all entities with whom you have this relationship or indicate none (add rows as needed)	Specifications/Comments (e.g., if payments were made to you or to your institution)
**10**	Leadership or fiduciary role in other board, society, committee or advocacy group, paid or unpaid	☒ **None**	
**11**	Stock or stock options	☒ **None**	
**12**	Receipt of equipment, materials, drugs, medical writing, gifts or other services	☒ **None**	
**13**	Other financial or non-financial interests	☒ **None**	

Please place an "X" next to the following statement to indicate your agreement:

☒ I certify that I have answered every question and have not altered the wording of any of the questions on this form.

ICMJE DISCLOSURE FORM

Date: 3/4/2022

Your Name: Robert Dobbins

Manuscript Title: Modelling Buprenorphine Reduction of Fentanyl-Induced Respiratory Depression

Manuscript Number (if known): 156973-INS-CMED-RV-3

In the interest of transparency, we ask you to disclose all relationships/activities/interests listed below that are related to the content of your manuscript. "Related" means any relation with for-profit or not-for-profit third parties whose interests may be affected by the content of the manuscript. Disclosure represents a commitment to transparency and does not necessarily indicate a bias. If you are in doubt about whether to list a relationship/activity/interest, it is preferable that you do so.

The author's relationships/activities/interests should be defined broadly. For example, if your manuscript pertains to the epidemiology of hypertension, you should declare all relationships with manufacturers of antihypertensive medication, even if that medication is not mentioned in the manuscript.

In item #1 below, report all support for the work reported in this manuscript without time limit. For all other items, the time frame for disclosure is the past 36 months.

		**Name all entities with whom you have this relationship or indicate none (add rows as needed)**	**Specifications/Comments (e.g., if payments were made to you or to your institution)**
**Time frame: Since the initial planning of the work**			
1	All support for the present manuscript (e.g., funding, provision of study materials, medical writing, article processing charges, etc.) **No time limit for this item.**	☐ **None**	
		Indivior Inc.	This study was supported by Indivior Inc., North Chesterfield, VA. The funder of the study and affiliated authors participated in designing the study, data analysis, and interpretation of the data. All authors had full access to all the data and agreed to submit the manuscript for publication.
			Click the tab key to add additional rows.
**Time frame: past 36 months**			
**2**	Grants or contracts from any entity (if not indicated in item #1 above).	☒ **None**	
**3**	Royalties or licenses	☒ **None**	

		Name all entities with whom you have this relationship or indicate none (add rows as needed)	Specifications/Comments (e.g., if payments were made to you or to your institution)
4	Consulting fees	☒ None	
5	Payment or honoraria for lectures, presentations, speakers bureaus, manuscript writing or educational events	☒ None	
6	Payment for expert testimony	☒ None	
7	Support for attending meetings and/or travel	☒ None	
8	Patents planned, issued or pending	☒ None	
9	Participation on a Data Safety Monitoring Board or Advisory Board	☒ None	
10	Leadership or fiduciary role in other board, society, committee or advocacy group, paid or unpaid	☒ None	

		Name all entities with whom you have this relationship or indicate none (add rows as needed)	Specifications/Comments (e.g., if payments were made to you or to your institution)
**11**	Stock or stock options	☒ **None**	
**12**	Receipt of equipment, materials, drugs, medical writing, gifts or other services	☒ **None**	
**13**	Other financial or non-financial interests	☐ **None**	
		Indivior Inc.	Employee - The funder provided support in the form of salaries for author.

Please place an "X" next to the following statement to indicate your agreement:

☒ I certify that I have answered every question and have not altered the wording of any of the questions on this form.

ICMJE DISCLOSURE FORM

**Date:**	3/7/2022
**Your Name:**	Geert Jan Groeneveld
**Manuscript Title:**	Modelling Buprenorphine Reduction of Fentanyl-Induced Respiratory Depression
**Manuscript Number (if known):**	156973-INS-CMED-RV-3

In the interest of transparency, we ask you to disclose all relationships/activities/interests listed below that are related to the content of your manuscript. "Related" means any relation with for-profit or not-for-profit third parties whose interests may be affected by the content of the manuscript. Disclosure represents a commitment to transparency and does not necessarily indicate a bias. If you are in doubt about whether to list a relationship/activity/interest, it is preferable that you do so.

The author's relationships/activities/interests should be defined broadly. For example, if your manuscript pertains to the epidemiology of hypertension, you should declare all relationships with manufacturers of antihypertensive medication, even if that medication is not mentioned in the manuscript.

In item #1 below, report all support for the work reported in this manuscript without time limit. For all other items, the time frame for disclosure is the past 36 months.

		**Name all entities with whom you have this relationship or indicate none (add rows as needed)**	**Specifications/Comments (e.g., if payments were made to you or to your institution)**
		**Time frame: Since the initial planning of the work**	
1	All support for the present manuscript (e.g., funding, provision of study materials, medical writing, article processing charges, etc.) **No time limit for this item.**	☐ None	
		Indivior Inc.	This study was supported by Indivior Inc., North Chesterfield, VA. The funder of the study and affiliated authors participated in designing the study, data analysis, and interpretation of the data. All authors had full access to all the data and agreed to submit the manuscript for publication.
			Click the tab key to add additional rows.
		**Time frame: past 36 months**	
2	Grants or contracts from any entity (if not indicated in item #1 above).	☒ **None**	
3	Royalties or licenses	☒ **None**	

		Name all entities with whom you have this relationship or indicate none (add rows as needed)	Specifications/Comments (e.g., if payments were made to you or to your institution)
**4**	Consulting fees	☒ **None**	
**5**	Payment or honoraria for lectures, presentations, speakers bureaus, manuscript writing or educational events	☒ **None**	
**6**	Payment for expert testimony	☒ **None**	
**7**	Support for attending meetings and/or travel	☒ **None**	
**8**	Patents planned, issued or pending	☒ **None**	
**9**	Participation on a Data Safety Monitoring Board or Advisory Board	☒ **None**	
**10**	Leadership or fiduciary role in other board, society, committee or advocacy group, paid or unpaid	☒ **None**	

		Name all entities with whom you have this relationship or indicate none (add rows as needed)	Specifications/Comments (e.g., if payments were made to you or to your institution)
**11**	Stock or stock options	☒ **None**	
**12**	Receipt of equipment, materials, drugs, medical writing, gifts or other services	☒ **None**	
**13**	Other financial or non-financial interests	☒ **None**	

Please place an "X" next to the following statement to indicate your agreement:

☒ I certify that I have answered every question and have not altered the wording of any of the questions on this form.

ICMJE DISCLOSURE FORM

Date: 3/4/2022

Your Name: Celine Laffont

Manuscript Title: Modelling Buprenorphine Reduction of Fentanyl-Induced Respiratory Depression

Manuscript Number (if known): 156973-INS-CMED-RV-3

In the interest of transparency, we ask you to disclose all relationships/activities/interests listed below that are related to the content of your manuscript. "Related" means any relation with for-profit or not-for-profit third parties whose interests may be affected by the content of the manuscript. Disclosure represents a commitment to transparency and does not necessarily indicate a bias. If you are in doubt about whether to list a relationship/activity/interest, it is preferable that you do so.

The author's relationships/activities/interests should be defined broadly. For example, if your manuscript pertains to the epidemiology of hypertension, you should declare all relationships with manufacturers of antihypertensive medication, even if that medication is not mentioned in the manuscript.

In item #1 below, report all support for the work reported in this manuscript without time limit. For all other items, the time frame for disclosure is the past 36 months.

		Name all entities with whom you have this relationship or indicate none (add rows as needed)	Specifications/Comments (e.g., if payments were made to you or to your institution)
**Time frame: Since the initial planning of the work**			
1	All support for the present manuscript (e.g., funding, provision of study materials, medical writing, article processing charges, etc.) **No time limit for this item.**	☐ None	
		Indivior Inc.	This study was supported by Indivior Inc., North Chesterfield, VA. The funder of the study and affiliated authors participated in designing the study, data analysis, and interpretation of the data. All authors had full access to all the data and agreed to submit the manuscript for publication.
			Click the tab key to add additional rows.
**Time frame: past 36 months**			
2	Grants or contracts from any entity (if not indicated in item #1 above).	☒ **None**	
3	Royalties or licenses	☒ **None**	

		Name all entities with whom you have this relationship or indicate none (add rows as needed)	Specifications/Comments (e.g., if payments were made to you or to your institution)
4	Consulting fees	☒ **None**	
5	Payment or honoraria for lectures, presentations, speakers bureaus, manuscript writing or educational events	☒ **None**	
6	Payment for expert testimony	☒ **None**	
7	Support for attending meetings and/or travel	☒ **None**	
8	Patents planned, issued or pending	☒ **None**	
9	Participation on a Data Safety Monitoring Board or Advisory Board	☒ **None**	
10	Leadership or fiduciary role in other board, society, committee or advocacy group, paid or unpaid	☒ **None**	

		Name all entities with whom you have this relationship or indicate none (add rows as needed)	Specifications/Comments (e.g., if payments were made to you or to your institution)
**11**	Stock or stock options	☒ **None**	
**12**	Receipt of equipment, materials, drugs, medical writing, gifts or other services	☒ **None**	
**13**	Other financial or non-financial interests	☐ **None**	
		Indivior Inc.	Employee - The funder provided support in the form of salaries for author.

Please place an "X" next to the following statement to indicate your agreement:

☒ I certify that I have answered every question and have not altered the wording of any of the questions on this form.

ICMJE DISCLOSURE FORM

**Date:**	3/4/2022
**Your Name:**	Laurence Moss
**Manuscript Title:**	Modelling Buprenorphine Reduction of Fentanyl-Induced Respiratory Depression
**Manuscript Number (if known):**	156973-INS-CMED-RV-3

In the interest of transparency, we ask you to disclose all relationships/activities/interests listed below that are related to the content of your manuscript. "Related" means any relation with for-profit or not-for-profit third parties whose interests may be affected by the content of the manuscript. Disclosure represents a commitment to transparency and does not necessarily indicate a bias. If you are in doubt about whether to list a relationship/activity/interest, it is preferable that you do so.

The author's relationships/activities/interests should be defined broadly. For example, if your manuscript pertains to the epidemiology of hypertension, you should declare all relationships with manufacturers of antihypertensive medication, even if that medication is not mentioned in the manuscript.

In item #1 below, report all support for the work reported in this manuscript without time limit. For all other items, the time frame for disclosure is the past 36 months.

		**Name all entities with whom you have this relationship or indicate none (add rows as needed)**	**Specifications/Comments (e.g., if payments were made to you or to your institution)**
		Time frame: Since the initial planning of the work	
1	All support for the present manuscript (e.g., funding, provision of study materials, medical writing, article processing charges, etc.)<br>No time limit for this item.	☐ None	
		Indivior Inc.	This study was supported by Indivior Inc., North Chesterfield, VA. The funder of the study and affiliated authors participated in designing the study, data analysis, and interpretation of the data. All authors had full access to all the data and agreed to submit the manuscript for publication.
			Click the tab key to add additional rows.
		Time frame: past 36 months	
2	Grants or contracts from any entity (if not indicated in item #1 above).	☒ **None**	
3	Royalties or licenses	☒ **None**	

		Name all entities with whom you have this relationship or indicate none (add rows as needed)	Specifications/Comments (e.g., if payments were made to you or to your institution)
4	Consulting fees	☒ None	
5	Payment or honoraria for lectures, presentations, speakers bureaus, manuscript writing or educational events	☒ None	
6	Payment for expert testimony	☒ None	
7	Support for attending meetings and/or travel	☒ None	
8	Patents planned, issued or pending	☒ None	
9	Participation on a Data Safety Monitoring Board or Advisory Board	☒ None	
10	Leadership or fiduciary role in other board, society, committee or advocacy group, paid or unpaid	☒ None	

		Name all entities with whom you have this relationship or indicate none (add rows as needed)	Specifications/Comments (e.g., if payments were made to you or to your institution)
**11**	Stock or stock options	☒ **None**	
**12**	Receipt of equipment, materials, drugs, medical writing, gifts or other services	☒ **None**	
**13**	Other financial or non-financial interests	☒ **None**	

Please place an "X" next to the following statement to indicate your agreement:

☒ I certify that I have answered every question and have not altered the wording of any of the questions on this form.

ICMJE DISCLOSURE FORM

Date: 3/7/2022

Your Name: Marieke Niesters

Manuscript Title: Modelling Buprenorphine Reduction of Fentanyl-Induced Respiratory Depression

Manuscript Number (if known): 156973-INS-CMED-RV-3

In the interest of transparency, we ask you to disclose all relationships/activities/interests listed below that are related to the content of your manuscript. "Related" means any relation with for-profit or not-for-profit third parties whose interests may be affected by the content of the manuscript. Disclosure represents a commitment to transparency and does not necessarily indicate a bias. If you are in doubt about whether to list a relationship/activity/interest, it is preferable that you do so.

The author's relationships/activities/interests should be defined broadly. For example, if your manuscript pertains to the epidemiology of hypertension, you should declare all relationships with manufacturers of antihypertensive medication, even if that medication is not mentioned in the manuscript.

In item #1 below, report all support for the work reported in this manuscript without time limit. For all other items, the time frame for disclosure is the past 36 months.

		Name all entities with whom you have this relationship or indicate none (add rows as needed)	Specifications/Comments (e.g., if payments were made to you or to your institution)
**Time frame: Since the initial planning of the work**			
1	All support for the present manuscript (e.g., funding, provision of study materials, medical writing, article processing charges, etc.) **No time limit for this item.**	☐ **None**	
		Indivior Inc.	This study was supported by Indivior Inc., North Chesterfield, VA. The funder of the study and affiliated authors participated in designing the study, data analysis, and interpretation of the data. All authors had full access to all the data and agreed to submit the manuscript for publication.
			Click the tab key to add additional rows.
**Time frame: past 36 months**			
2	Grants or contracts from any entity (if not indicated in item #1 above).	☒ **None**	
3	Royalties or licenses	☒ **None**	

		Name all entities with whom you have this relationship or indicate none (add rows as needed)	Specifications/Comments (e.g., if payments were made to you or to your institution)
**4**	Consulting fees	☒ **None**	
**5**	Payment or honoraria for lectures, presentations, speakers bureaus, manuscript writing or educational events	☒ **None**	
**6**	Payment for expert testimony	☒ **None**	
**7**	Support for attending meetings and/or travel	☒ **None**	
**8**	Patents planned, issued or pending	☒ **None**	
**9**	Participation on a Data Safety Monitoring Board or Advisory Board	☒ **None**	
**10**	Leadership or fiduciary role in other board, society, committee or advocacy group, paid or unpaid	☒ **None**	

		Name all entities with whom you have this relationship or indicate none (add rows as needed)	Specifications/Comments (e.g., if payments were made to you or to your institution)
**11**	Stock or stock options	☒ **None**	
**12**	Receipt of equipment, materials, drugs, medical writing, gifts or other services	☒ **None**	
**13**	Other financial or non-financial interests	☒ **None**	

Please place an "X" next to the following statement to indicate your agreement:

☒ I certify that I have answered every question and have not altered the wording of any of the questions on this form.

ICMJE DISCLOSURE FORM

Date: 3/4/2022

Your Name: Erik Olofsen

Manuscript Title: Modelling Buprenorphine Reduction of Fentanyl-Induced Respiratory Depression

Manuscript Number (if known): 156973-INS-CMED-RV-3

In the interest of transparency, we ask you to disclose all relationships/activities/interests listed below that are related to the content of your manuscript. "Related" means any relation with for-profit or not-for-profit third parties whose interests may be affected by the content of the manuscript. Disclosure represents a commitment to transparency and does not necessarily indicate a bias. If you are in doubt about whether to list a relationship/activity/interest, it is preferable that you do so.

The author's relationships/activities/interests should be defined broadly. For example, if your manuscript pertains to the epidemiology of hypertension, you should declare all relationships with manufacturers of antihypertensive medication, even if that medication is not mentioned in the manuscript.

In item #1 below, report all support for the work reported in this manuscript without time limit. For all other items, the time frame for disclosure is the past 36 months.

		Name all entities with whom you have this relationship or indicate none (add rows as needed)	Specifications/Comments (e.g., if payments were made to you or to your institution)
**Time frame: Since the initial planning of the work**			
1	All support for the present manuscript (e.g., funding, provision of study materials, medical writing, article processing charges, etc.) **No time limit for this item.**	☐ None	
		Indivior Inc.	This study was supported by Indivior Inc., North Chesterfield, VA. The funder of the study and affiliated authors participated in designing the study, data analysis, and interpretation of the data. All authors had full access to all the data and agreed to submit the manuscript for publication.
			Click the tab key to add additional rows.
**Time frame: past 36 months**			
2	Grants or contracts from any entity (if not indicated in item #1 above).	☒ **None**	
3	Royalties or licenses	☒ **None**	

		Name all entities with whom you have this relationship or indicate none (add rows as needed)	Specifications/Comments (e.g., if payments were made to you or to your institution)
**4**	Consulting fees	☒ **None**	
**5**	Payment or honoraria for lectures, presentations, speakers bureaus, manuscript writing or educational events	☒ **None**	
**6**	Payment for expert testimony	☒ **None**	
**7**	Support for attending meetings and/or travel	☒ **None**	
**8**	Patents planned, issued or pending	☒ **None**	
**9**	Participation on a Data Safety Monitoring Board or Advisory Board	☒ **None**	
**10**	Leadership or fiduciary role in other board, society, committee or advocacy group, paid or unpaid	☒ **None**	

		Name all entities with whom you have this relationship or indicate none (add rows as needed)	Specifications/Comments (e.g., if payments were made to you or to your institution)
**11**	Stock or stock options	☒ **None**	
**12**	Receipt of equipment, materials, drugs, medical writing, gifts or other services	☒ **None**	
**13**	Other financial or non-financial interests	☒ **None**	

Please place an "X" next to the following statement to indicate your agreement:

☒ I certify that I have answered every question and have not altered the wording of any of the questions on this form.

ICMJE DISCLOSURE FORM

Date: 3/7/2022

Your Name: Monique van Velzen

Manuscript Title: Modelling Buprenorphine Reduction of Fentanyl-Induced Respiratory Depression

Manuscript Number (if known): 156973-INS-CMED-RV-3

In the interest of transparency, we ask you to disclose all relationships/activities/interests listed below that are related to the content of your manuscript. "Related" means any relation with for-profit or not-for-profit third parties whose interests may be affected by the content of the manuscript. Disclosure represents a commitment to transparency and does not necessarily indicate a bias. If you are in doubt about whether to list a relationship/activity/interest, it is preferable that you do so.

The author's relationships/activities/interests should be defined broadly. For example, if your manuscript pertains to the epidemiology of hypertension, you should declare all relationships with manufacturers of antihypertensive medication, even if that medication is not mentioned in the manuscript.

In item #1 below, report all support for the work reported in this manuscript without time limit. For all other items, the time frame for disclosure is the past 36 months.

		Name all entities with whom you have this relationship or indicate none (add rows as needed)	Specifications/Comments (e.g., if payments were made to you or to your institution)
		**Time frame: Since the initial planning of the work**	
1	All support for the present manuscript (e.g., funding, provision of study materials, medical writing, article processing charges, etc.) **No time limit for this item.**	☐ None	
		Indivior Inc.	This study was supported by Indivior Inc., North Chesterfield, VA. The funder of the study and affiliated authors participated in designing the study, data analysis, and interpretation of the data. All authors had full access to all the data and agreed to submit the manuscript for publication.
			Click the tab key to add additional rows.
		**Time frame: past 36 months**	
2	Grants or contracts from any entity (if not indicated in item #1 above).	☒ **None**	
3	Royalties or licenses	☒ **None**	

		Name all entities with whom you have this relationship or indicate none (add rows as needed)	Specifications/Comments (e.g., if payments were made to you or to your institution)
**4**	Consulting fees	☒ **None**	
**5**	Payment or honoraria for lectures, presentations, speakers bureaus, manuscript writing or educational events	☒ **None**	
**6**	Payment for expert testimony	☒ **None**	
**7**	Support for attending meetings and/or travel	☒ **None**	
**8**	Patents planned, issued or pending	☒ **None**	
**9**	Participation on a Data Safety Monitoring Board or Advisory Board	☒ **None**	
**10**	Leadership or fiduciary role in other board, society, committee or advocacy group, paid or unpaid	☒ **None**	

		Name all entities with whom you have this relationship or indicate none (add rows as needed)	Specifications/Comments (e.g., if payments were made to you or to your institution)
**11**	Stock or stock options	☒ **None**	
**12**	Receipt of equipment, materials, drugs, medical writing, gifts or other services	☒ **None**	
**13**	Other financial or non-financial interests	☒ **None**	

Please place an "X" next to the following statement to indicate your agreement:

☒ I certify that I have answered every question and have not altered the wording of any of the questions on this form.

JCI The Journal of Clinical Investigation

Click on boxes to check/uncheck items.

Checklist for submitting a revised Clinical Medicine manuscript

In addition to addressing the items noted in the decision letter regarding your manuscript, ensure that your revised manuscript adheres to the guidelines below. For full submission details, visit the JCI website.

Required files

Manuscript

- ☑ PDF of a clean version of the entire manuscript, including figures, figure legends, and tables
- ☑ PDF of a marked-up version of the entire manuscript showing revisions and prefaced by a point-by-point response to reviewer comments
- ☑ Word or RTF file of all text of the submission, including Figure legends, Tables, Table legends, and References (without Figures, images, or point-by-point responses)
- ☑ Single PDF file of completed ICMJE uniform disclosure forms from all authors
- ☐ For clinical trials, PDF of the appropriate reporting checklist (CONSORT, STROBE. etc.)

Figures

- ☐ Publication-quality figures in TIFF format. See detailed instructions for figure preparation.
- ☐ Recommended: Graphical abstract (details available here)

Supplemental material

- ☑ Single file containing supplemental material, figures, and modest-sized tables, as:
  - ☐ (a) (if applicable) a PDF highlighting reviewer-requested changes
  - ☑ (b) a clean, publication-quality PDF
- ☐ Upload any supplemental videos and/or large Excel files separately

Gels

- ☐ APDF, PPT, or PPTX file (distinct from any other supplemental material) that shows the entire unedited gel
- ☐ Clearly indicate which bands were used for the figures

Formatting

- ☑ Recommended 9,,000/maximum 12,000 words (all text inclusive of Title page, Full text, References, Figure legends, and Tables)
- ☑ Double-spacing throughout, including references and tables; figure legends may be single spaced if necessary to keep a figure and its legend on the same page
- ☑ All pages numbered
- ☑ Each section begins on a new page

Abbreviations and acronyms

- ☑ Standard JCI abbreviations and acronyms used without definition
- ☑ All other abbreviations and acronyms spelled out at first use in the Abstract and again at first use in the main text (with the abbreviated form appearing in parentheses), and used without definition thereafter

Gene and protein names and symbols

- ☑ Conform to official NCBI Gene Nomenclature
- ☑ Presented according to JCI Gene nomenclature and style

Italicization

- ☑ Generally reserved for gene symbols, genotypes, and species names
- ☑ Terms such as in vivo, in vitro, etc., are not italicized

Unpublished data, manuscripts in preparation or under review, and personal communications

- ☑ Cited parenthetically in the text, not as numbered references; e.g., "(Jane L. Doe, UCLA, Los Angeles, California, USA, unpublished observations)"
- ☑ Written permission to cite unpublished observations of someone outside the author's research team (an email is sufficient) is submitted

Reference citations

- ☑ Appear in parentheses preceded by a space, e.g., "as described previously (1, 2)"; "several research groups (4–10) have found"
- ☑ No superscript, bold, italics, etc.

Figure and table callouts

- ☑ Figures and tables called out in numerical order
- ☑ "Figure", "Table", "Supplemental Figure", "Supplemental Table", etc., spelled out
- ☑ Callouts in parentheses (no boldface or italics) unless grammatically part of the sentence: "the levels increased (Figure 5A)"; "data shown in Table 2"
- ☑ Parts called out as follows: "Figure 1A", "Figure 2, A and B", "Figure 3, B–D"

Manuscript preparation and required reporting

☑ Title page

☑ Manuscript title

- ☑ Clear, concise, and limited to 15 words, including conjunctions
- ☑ Refers to the relevant disease or disease model studied
- ☑ No subtitles, colons, periods, or nonstandard abbreviations

☑ Authors and affiliations

- ☑ Author names provided in full (for example, "Benita J. Sjögren") and in the appropriate order
- ☑ No titles, honorifics, degrees, or certifications
- ☑ Author affiliations correspond to the period when the work was performed
- ☐ For authors whose affiliation has changed since completion of the work, specify the present affiliation and location below the numbered list
- ☑ Affiliation footnotes assigned consecutively using superscripted numbers (1, 2, 3, etc.)
- ☑ Affiliations include departments, institutions, city, state (if applicable), and country (but not mailing addresses or zip/regional codes)
- ☑ Corresponding author's complete name, address, telephone number (including country code if applicable), and email address
- ☑ Consortium/study groups shown as authors (e.g., CARDIoGRAM Consortium)
  - ☑ Unless the members of the group appear as authors, each individual member and their affiliation are listed in the supplemental material, under the heading Supplemental Acknowledgments
  - ☑ The following sentence appears in Acknowledgments: "See Supplemental Acknowledgments for details on {name of consortium}."

☑ Conflict-of-interest statement

- ☑ A statement consistent with the Journal's conflict-of-interest policy is included; if no author has a conflict, state the following: "The authors have declared that no conflict of interest exists."
- ☐ If patents are involved, the patent or patent application number(s) are provided, and the names of the associated authors specified

Created August, updated October 2021

☑ IAbstract

☑ Structured format with the sections Background, Methods, Results, Conclusion, Trial registration, Funding

☑ Maximum 250 words

☑ No references

☑ All nonstandard abbreviations defined at first use

Main text (presented in the following order)

☑ Introduction

☑ Results

☑ Discussion

☑ Methods

☑ Demographic reporting

- ☐ Reporting on race and ethnicity adheres to NIH guidelines or other applicable authoritative standards
- ☑ Descriptors for any demographic identities are clear, unbiased, and up-to-date
- ☑ Data for any demographic variable are inclusive; if any information is unavailable or incomplete, an explanation is provided
- ☐ Specify whether the participants or investigators made the classifications; and whether the options were defined by the investigators or participants

☑ Complete manufacturer name (omit location) provided for each proprietary item used

☑ For animal models, precise genotype, strain, number of backcrosses, sex, age, and source are specifed

☑ Description of all antibodies used, including the source and catalog/clone number for commercial antibodies or (reference to) a description of the generation of custom antibodies

☑ Source of all cell lines used is indicated

☑ Data sets for gene expression microarrays, SNP arrays, and high-throughput sequencing studies are deposited in a public repository, and accession number(s) provided in Methods the main text (for publication, data must be publicly available)

☑ Statistics

- ☑ Section appears near the end of Methods (before "Study approval")
- ☑ The *P* value used to determine significance is specified; e.g., "A *P* value less than 0.05 was considered significant."
- ☑ Analysis appropriately corrects for multiple comparisons (more than 2 groups) and for repeated measures (multiple measurements within subjects)
- ☑ If samples were excluded, a statement describes inclusion/exclusion criteria

☑ Study approval

- ☑ Stand-alone paragraph at the end of Methods
- ☑ Declaration of approval of human and/or animal studies, specifying the name and location of the appropriate institutional review board(s)
- ☑ For human studies, a statement indicating receipt of written informed consent from participants and/or their parents/guardians
- ☑ For use of photographs of patients, a separate statement of written informed consent

☑ Author contributions

☑ Contribution of each author (identified by initials) is specified

☑ Grammatically complete sentences

☑ For manuscripts with 2 or more co–first authors, the method used to assign authorship order among these authors is stated

☐ Acknowledgments

☐ States sources of support in the form of grants, equipment, or drugs

☐ Grant numbers provided as applicable

☐ Other appropriate acknowledgments, such as of colleagues for advice

☑ References

☑ Styled according to Journal reference instructions

☑ Figure legends

☑ Maximum 300 words

☑ Each begins with stand-alone title, irrespective of the individual parts

☑ Figure parts called out in boldface: (**A**), (**B**–**D**), (**C** and **E**)

☑ Symbols and abbreviations introduced in figures are defined

☑ In each figure legend where appropriate, the statistical test(s) used is described

☐ Variance around the mean and statistical analysis not provided for figures representing fewer than 3 independent samples

☐ For figure panels representing multiple experiments, exact number of samples (*n*) is reported

☐ For representative experiments, the number of times the experiment was conducted is reported

☑ For histological panels and insets, scale bars are defined or total original magnification is specified in the figure legends

☑ Figures

☑ Prepared according to Journal figure instructions

☑ For clinical trials, the appropriate flow diagram appears as a figure

☑ Parts labeled with capital letters: A, B, C, etc., with no designated subparts

☐ Graphs of quantitative data presented as either dot plots, with average and appropriate error bars indicated; or box-and-whisker plots, with values defined in the legend (bounds of the boxes, lines within the boxes, whiskers, and any outlying values); dynamite plunger plots are not permitted

☑ If lanes in a gel or blot image are spliced together into a composite image, the lanes are separated with a thin vertical line (black on gray background; white on black background); a note in the legend states that the lanes were run on the same gel but were noncontiguous

☑ Tables

☑ Prepared in Word table format (not pasted in from another application)

☑ Self-contained and self-explanatory

☑ Each table fits on a single page and is presented on its own page

☑ Preceded by brief titles

☑ Callouts to footnotes (designated with superscript capital letters) assigned alphabetically row by row

☑ No subparts or subsections (for example, Table 1A and Table 1B)

☑ Column headings in tables apply to all values throughout the column; a new row of column headings may not be introduced within a table

☑ See "Methods" above for demographics reporting

☑ Supplemental material

☑ A single PDF file includes all supplemental material except videos and spreadsheets. See "Methods" above for large data sets

☑ Before submission, carefully review all supplmental files; they will not be checked by a copy editor. The Journal is not responsible for any errors contained in supplemental material.
